# Supplementary figures and images for: The glucose-sensing transcription factor MLX balances metabolism and stress to suppress apoptosis and maintain spermatogenesis
Source: PLoS Biol. 2021 Oct 20;19(10):e3001085. doi: 10.1371/journal.pbio.3001085 (PMC8528285; doi:10.1371/journal.pbio.3001085)

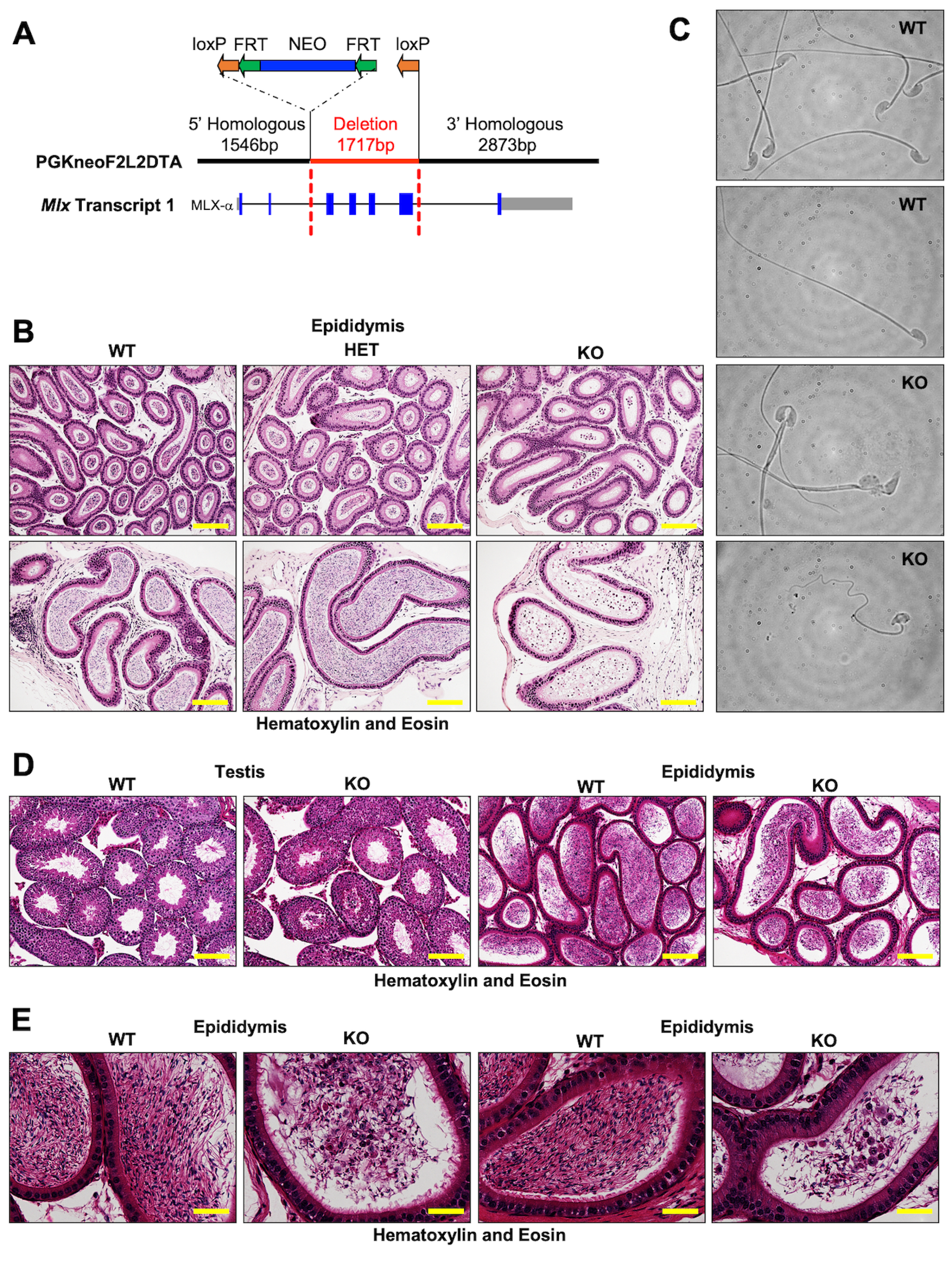

Supplement: S1 Fig — Histological characterization of the WT, HET, and MLXKO testes and epididymides. (A) Schematic of the targeting construct used to generate deletion of murine Mlx. (B) Histological analysis of WT, HET, and MLXKO epididymis stained with hematoxylin and eosin (100×, scale bar = 400 uM). (C) Images of cauda epididymal spermatozoa from WT versus MLXKO mice, the latter with typical abnormal features such as altered tail and head morphology. (D) Histological analysis of p51 WT versus MLXKO testis and epididymis stained with hematoxylin and eosin (100×, scale bar = 400 uM). (E) Histological analysis of p51 WT versus MLXKO epididymis stained with hematoxylin and eosin (400×, scale bar = 100 uM). HET, heterozygous; KO, knockout; MLX, MAX-Like protein X; WT, wild-type. (TIF) [file pbio.3001085.s001.tif]

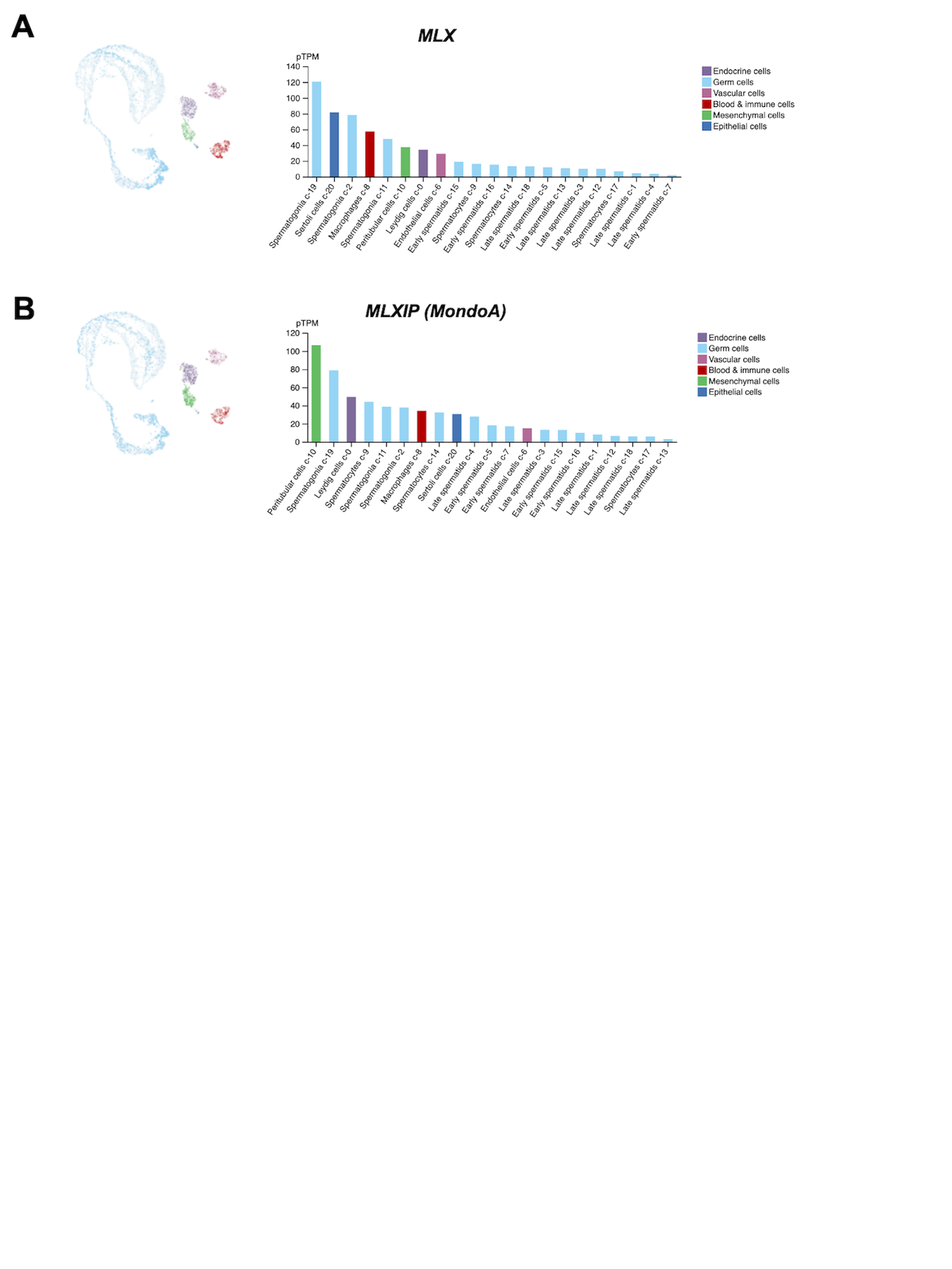

Supplement: S2 Fig — Widespread expression of MLX and MondoA transcripts in the human testes. The Human Protein Atlas database [22] scRNA-seq dataset from adult human testes Guo (2018) [23] was queried for testes expression of (A) MLX and (B) MLXIP (encoding MondoA). Both are present in multiple stromal and GC populations, with high expression in primitive GCs and Sertoli (MLX) and Peritubular Myoid cells (MondoA). Image credit: Human Protein Atlas. Image available from v20.1.proteinatlas.org (http://www.proteinatlas.org). The underlying data for S2A and S2B Fig can be found in S1 Data. GC, germ cell; MLX, MAX-Like protein X; scRNA-seq, single-cell RNA sequencing. (TIF) [file pbio.3001085.s002.tif]

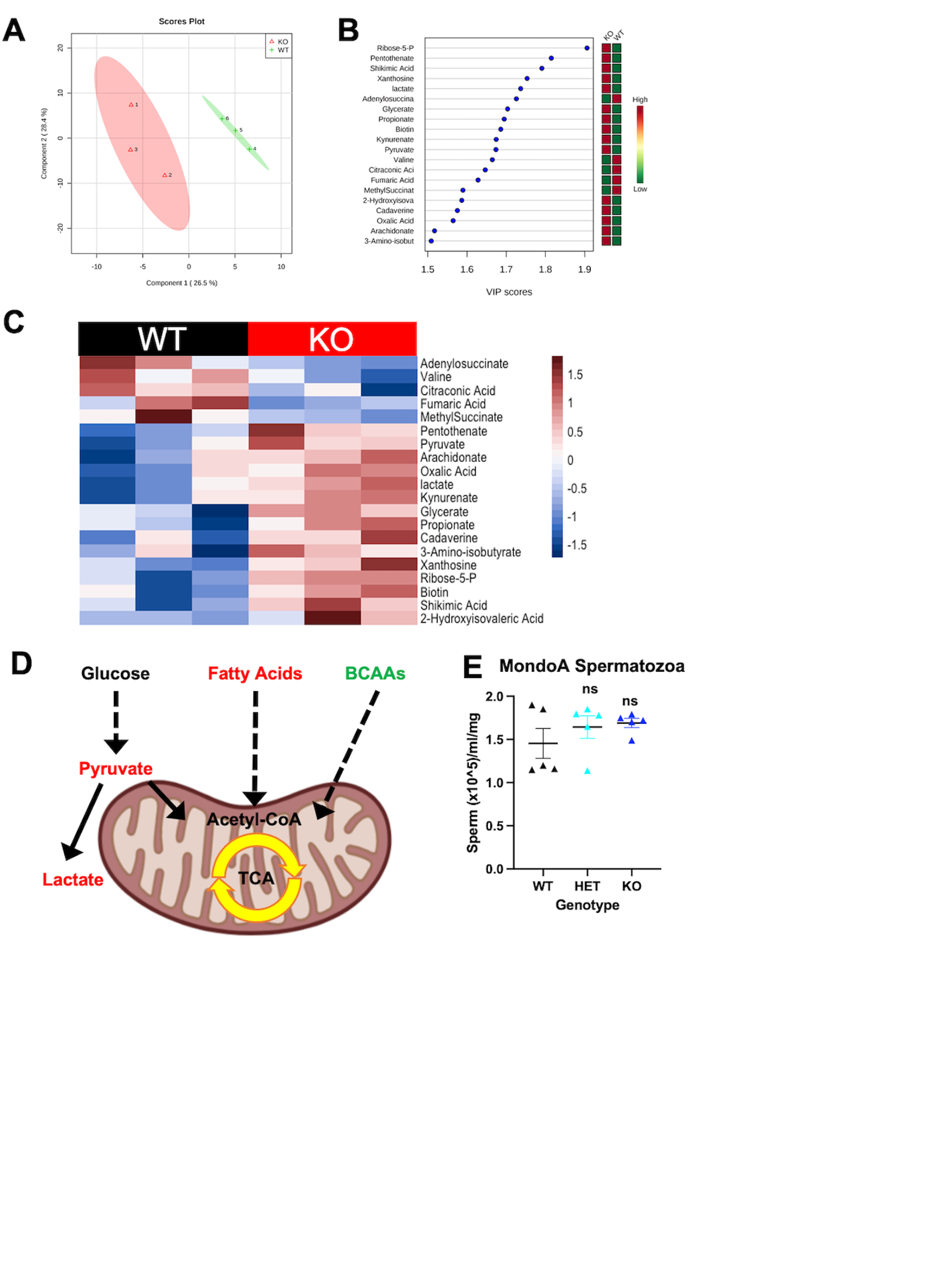

Supplement: S3 Fig — Metabolomic data from WT and MLXKO serum. (A) PLS-DA and (B) VIP plot from the metabolomic dataset analyzed by MetaboAnalyst 4.0 [25]. (C) Heat map of mean-centered serum metabolomics dataset from WT versus MLXKO mice showing the top 20 VIP features from PLS-DA (N = 3). (D) A model of altered mitochondrial fuel source based upon serum metabolomics (red indicates up in the MLXKO and green indicates down). Image made with BioRender. (E) Sperm count from MondoA WT versus KO mice (N = 5) tested with a paired t test. The underlying data for S3A–S3C and S3E Fig can be found in S1 Data. KO, knockout; MLX, MAX-Like protein X; PLS-DA, partial least squares discriminant analysis; VIP, variable importance to projection; WT, wild-type. (TIF) [file pbio.3001085.s003.tif]

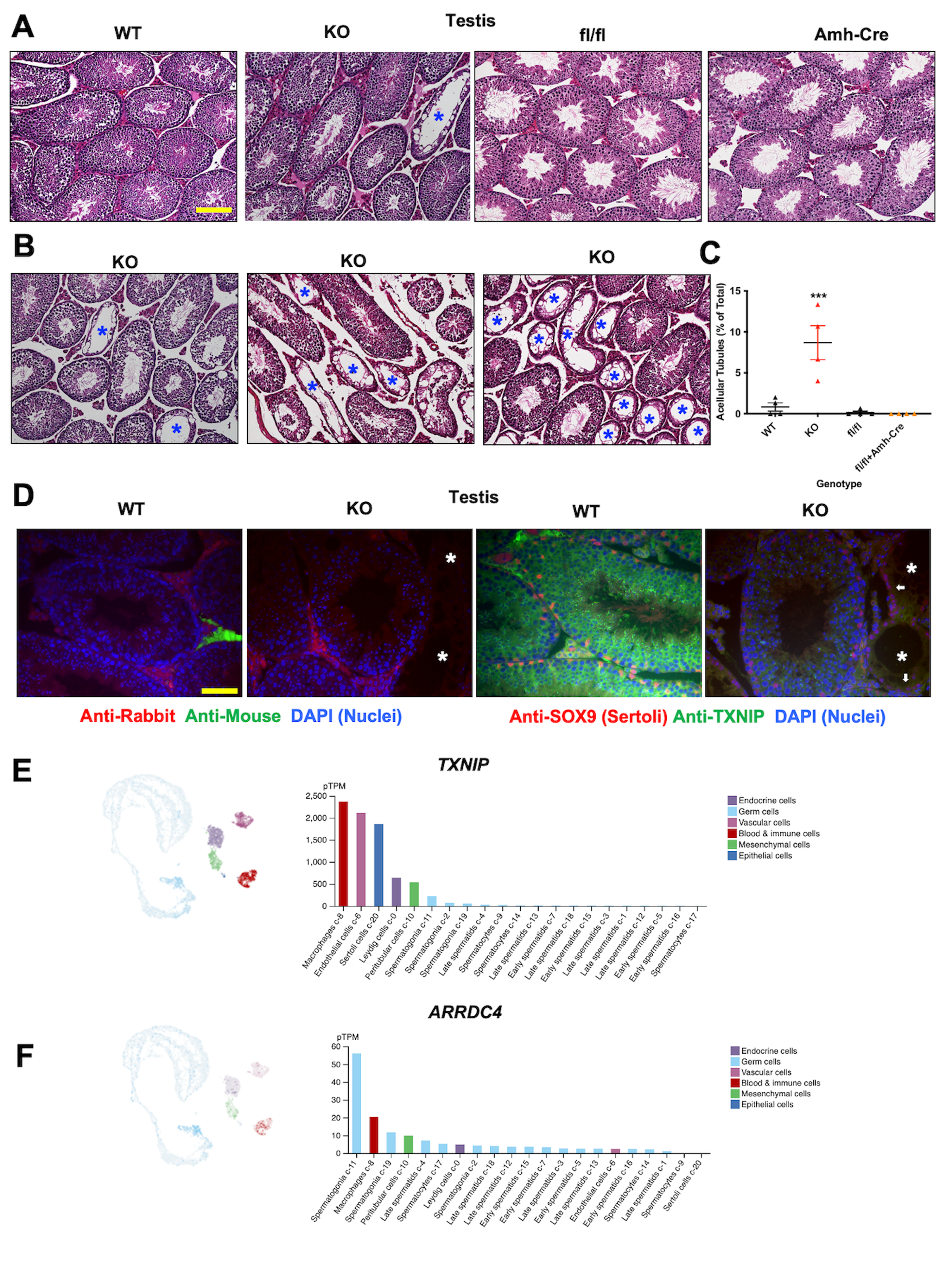

Supplement: S4 Fig — Comparison of seminiferous tubules and gene expression in WT and MLXKO testes. (A) Histological analysis of testes from 6-month-old males of the indicated genotype stained with hematoxylin and eosin (100×, scale bar = 400 uM). Asterisks mark acellular tubules (B) Staining, as in (A) demonstrating the range of acellular tubule frequency specific to the whole body MLX deletion. (C) Quantification of acellular tubules as the percentage of acellular tubules per 50 tubules (average of 250 total tubules per animal, 4 animals per genotype. Shown is the mean with SEM (* p < 0.05, ** p < 0.01, *** p < 0.001, *** p < 0.0001). (D) IF staining of WT and MLXKO testes tissue for TXNIP and SOX9. Asterisks marks and acellular tubule. Arrow indicates SOX9+ cells still present in the GC depleted tubule (200×, scale bar = 200 uM). (E, F). Human Protein Atlas [22] scRNA-seq dataset from adult human testes Guo 2018 [23] was queried for the established MLX targets: (E) TXNIP and (F) ARRDC4. Both are present in multiple stromal and GC populations. Image credit: Human Protein Atlas. Image available from v20.1.proteinatlas.org (http://www.proteinatlas.org). The underlying data for S4C, S4E, and S4F Fig can be found in S1 Data. GC, germ cell; IF, immunofluorescence; KO, knockout; MLX, MAX-Like protein X; scRNA-seq, single-cell RNA sequencing; WT, wild-type. (TIF) [file pbio.3001085.s004.tif]

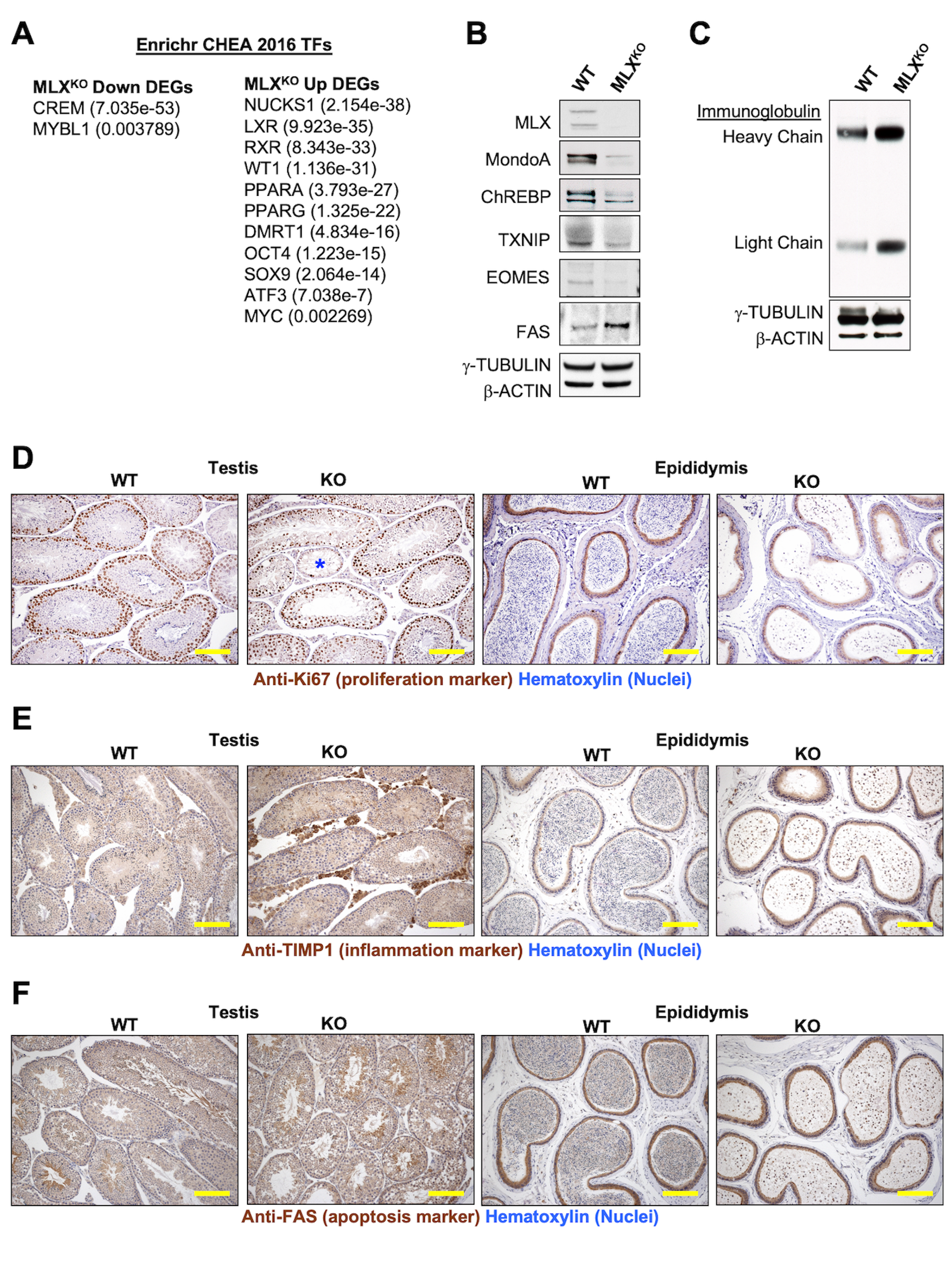

Supplement: S5 Fig — RNA profiling of testes from WT versus MLXKO mice. (A) CHEA (2016 from Enrichr database) adjusted p-values for indicated TF targets associated with up or down in the WT versus the MLXKO RNA-seq data. (B,C) WB data from whole testes lysates from WT versus MLXKO mice probed for the indicated proteins. (D) IHC analysis of WT versus MLXKO testis and epididymis stained for the proliferation marker Ki-67 (100×, scale bar = 400 uM). Asterisks marks acellular tubule with decreased Ki-67. (E) IHC analysis of WT versus MLXKO testis and epididymis stained for TIMP1 (100×, scale bar = 400 uM). (F) IHC analysis of WT versus MLXKO testis and epididymis stained for FAS (100×, scale bar = 400 uM). CHEA, ChIP set enrichment analysis; IHC, immunohistochemistry; KO, knockout; MLX, MAX-Like protein X; RNA-seq, RNA sequencing; TF, transcription factor; WB, western blot; WT, wild-type. (TIF) [file pbio.3001085.s005.tif]

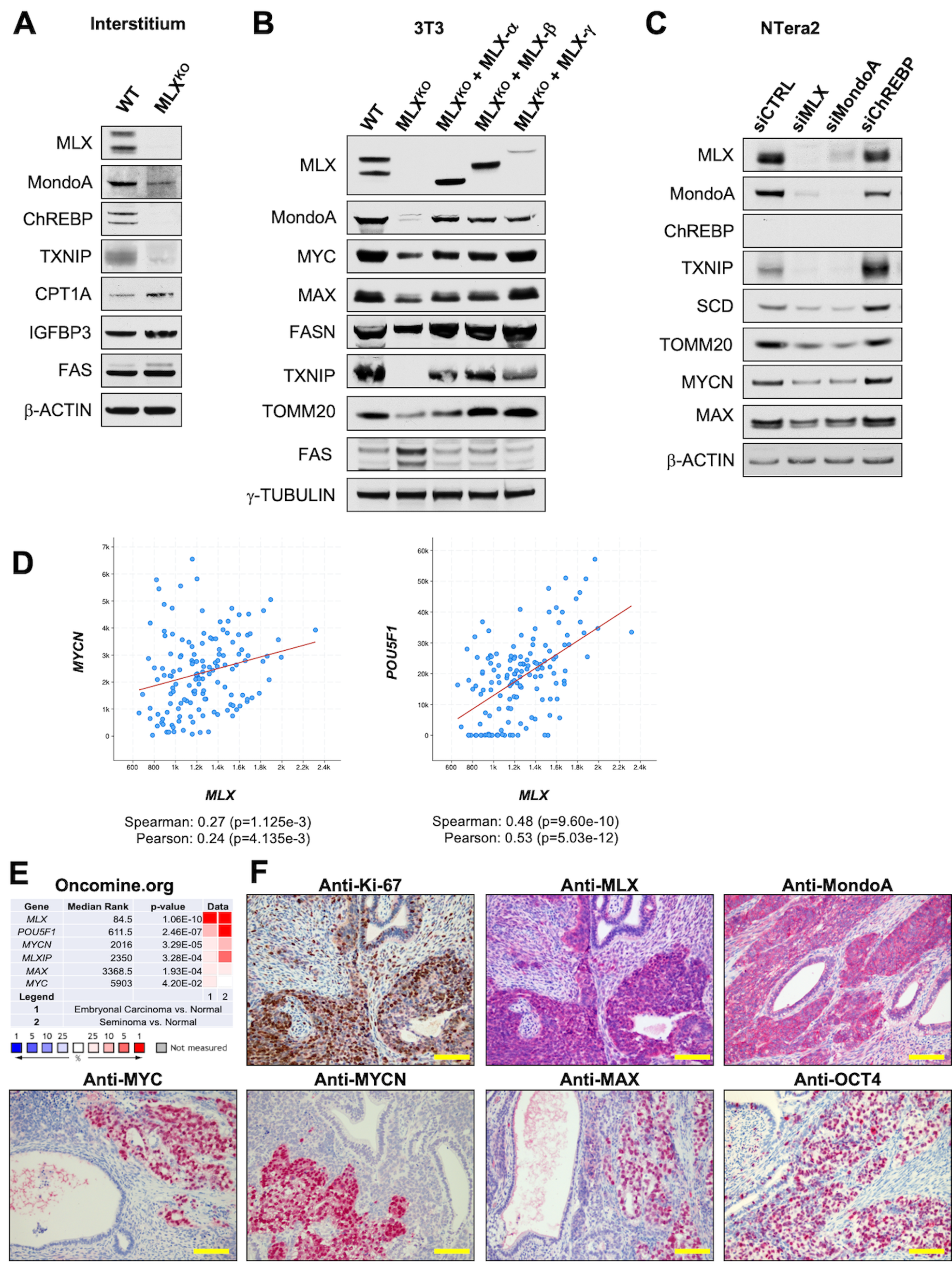

Supplement: S6 Fig — Molecular, biochemical, and functional validation of GSEA categories from WT versus MLXKO mice. (A) WB of cells isolated from the interstitium of WT versus MLXKO testes tissue probed for the indicated proteins. (B) WB analysis of the WT versus MLXKO 3T3 cell lines reconstituted with empty vector or the indicated isoform of MLX probed for the indicated proteins. (C) WB analysis of the NTera2 cells treated with the indicated siRNA, probed for the indicated proteins. (D) Co-expression analysis of MLX and POU5F1 from the Testicular Germ Cell Tumor Dataset (TCGA PanCancer Atlas). (E) Overexpression of the indicated mRNAs from Oncomine.org Korkola and colleagues dataset. (F) IHC analysis of NTera2 cell xenograft [45] stained for the indicated proteins (200×, scale bar = 200 uM). The underlying data for S6D and S6E Fig and can be found in S1 Data. GSEA, gene set enrichment analysis; IHC, immunohistochemistry; KO, knockout; siRNA, small interfering RNA; WB, western blot; WT, wild-type. (TIF) [file pbio.3001085.s006.tif]

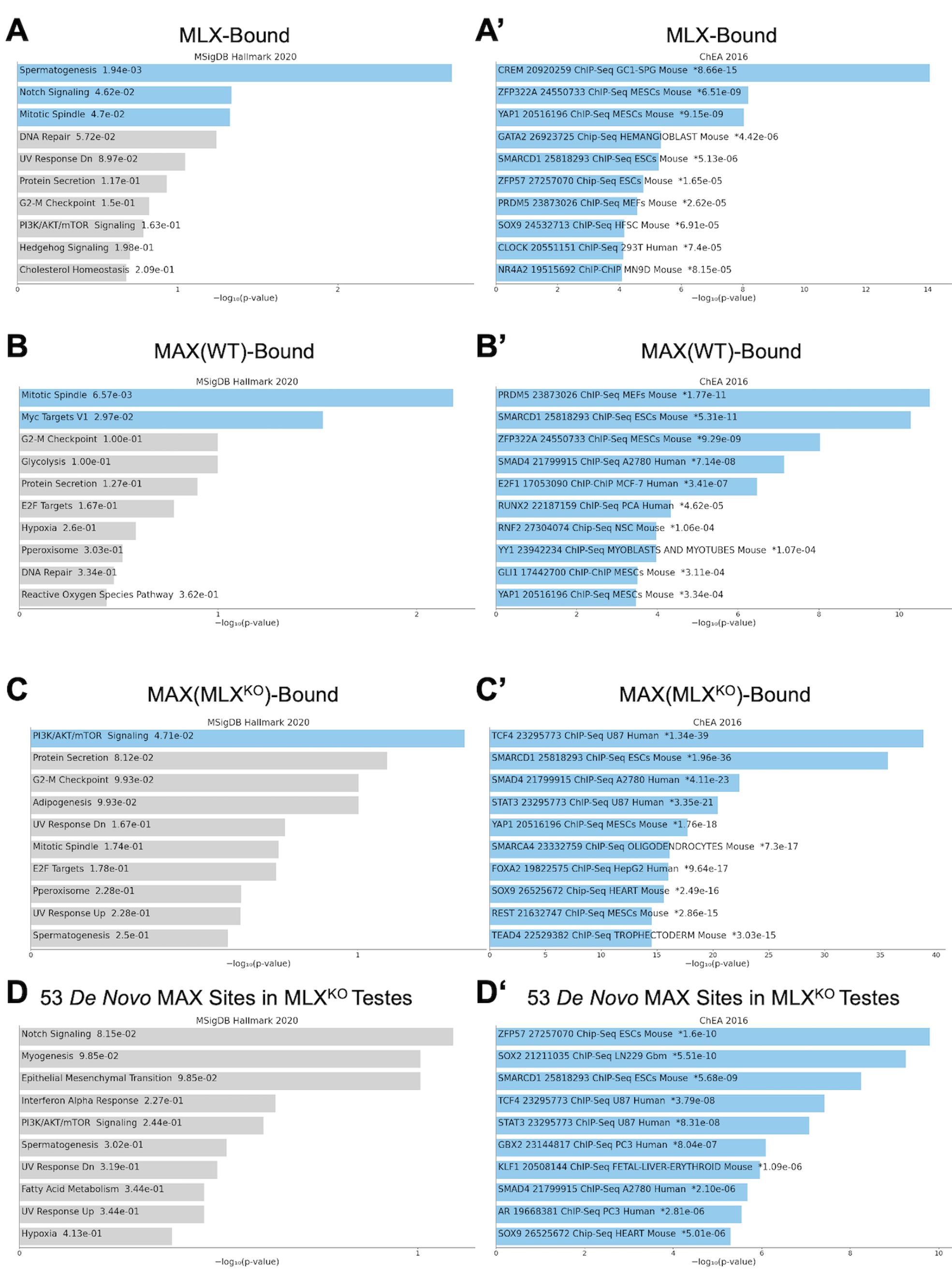

Supplement: S7 Fig — Enrichment analysis of MLX and MAX binding in the mouse testes. (A, A’) Enrichr analysis of genes bound by MLX (MLX bound) analyzed for enrichment of pathways by MSigDB (A) and transcriptional regulators by CHEA (A’). (B, B’). Enrichr analysis of genes bound by MAX in the presence of MLX, MAX(WT)-Bound analyzed for enrichment of MSigDB (B) and CHEA (B’). (C, C’) Enrichr analysis of genes bound by MAX in the absence of MLX, MAX(MLXKO)-Bound analyzed for enrichment of MSigDB (C) and CHEA (C’). (D) Enrichr analysis of genes previously bound by MLX, but only bound by MAX in the absence of MLX, de novo genes MAX(MLXKO)-Bound analyzed for enrichment of MSigDB (D) and CHEA (D’). All Enrichr images created with Appyter [98]. The underlying data for S7A–S7D Fig can be found in S1 Data. CHEA, ChIP set enrichment analysis; KO, knockout; MLX, MAX-Like protein X; MSigDB, Molecular Signature Database; WT, wild-type. (TIF) [file pbio.3001085.s007.tif]

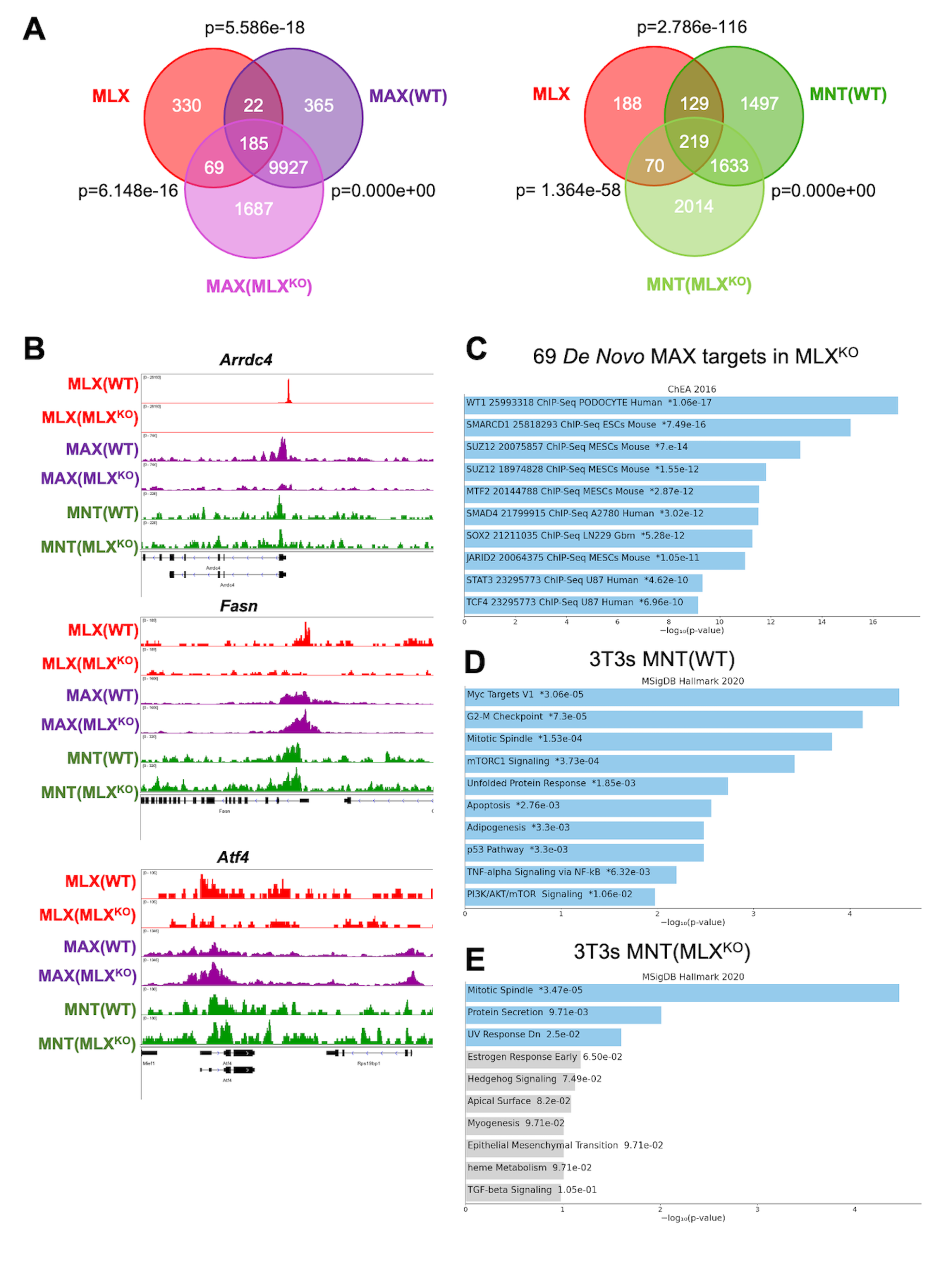

Supplement: S8 Fig — MLX shares numerous transcriptional targets with MAX and MNT. (A) Venn diagram of the overlap of genes bound by MLX with MAX (Left), and MLX with MNT (Right), in both WT and MLXKO 3T3 cells with p-value calculated from a hypergeometric test. (B) IGV tracks for MLX, MAX, and MNT on the Arrdc4, Fasn, and Atf4 promoters from WT and MLXKO 3T3 cells. (C) Enrichr analysis of the subset of MLX-bound and MAX-bound peaks that are only bound in the MLXKO 3T3, de novo MAX sites MAX(MLXKO)-Bound for CHEA enrichment. (D) Enrichr analysis of MNT-Bound genes only occupied in the presence of MLX, MNT(WT)-Bound for MsigDB enrichment. (E) Enrichr analysis of MNT-Bound genes only occupied in the absence of MLX, MNT(MLXKO)-Bound for MsigDB enrichment. All Enrichr images created with Appyter [98]. The underlying data for S8A and S8E Fig can be found in S1 Data. CHEA, ChIP set enrichment analysis; IGV, Integrative Genomics Viewer; KO, knockout; MLX, MAX-Like protein X; WT, wild-type. (TIF) [file pbio.3001085.s008.tif]

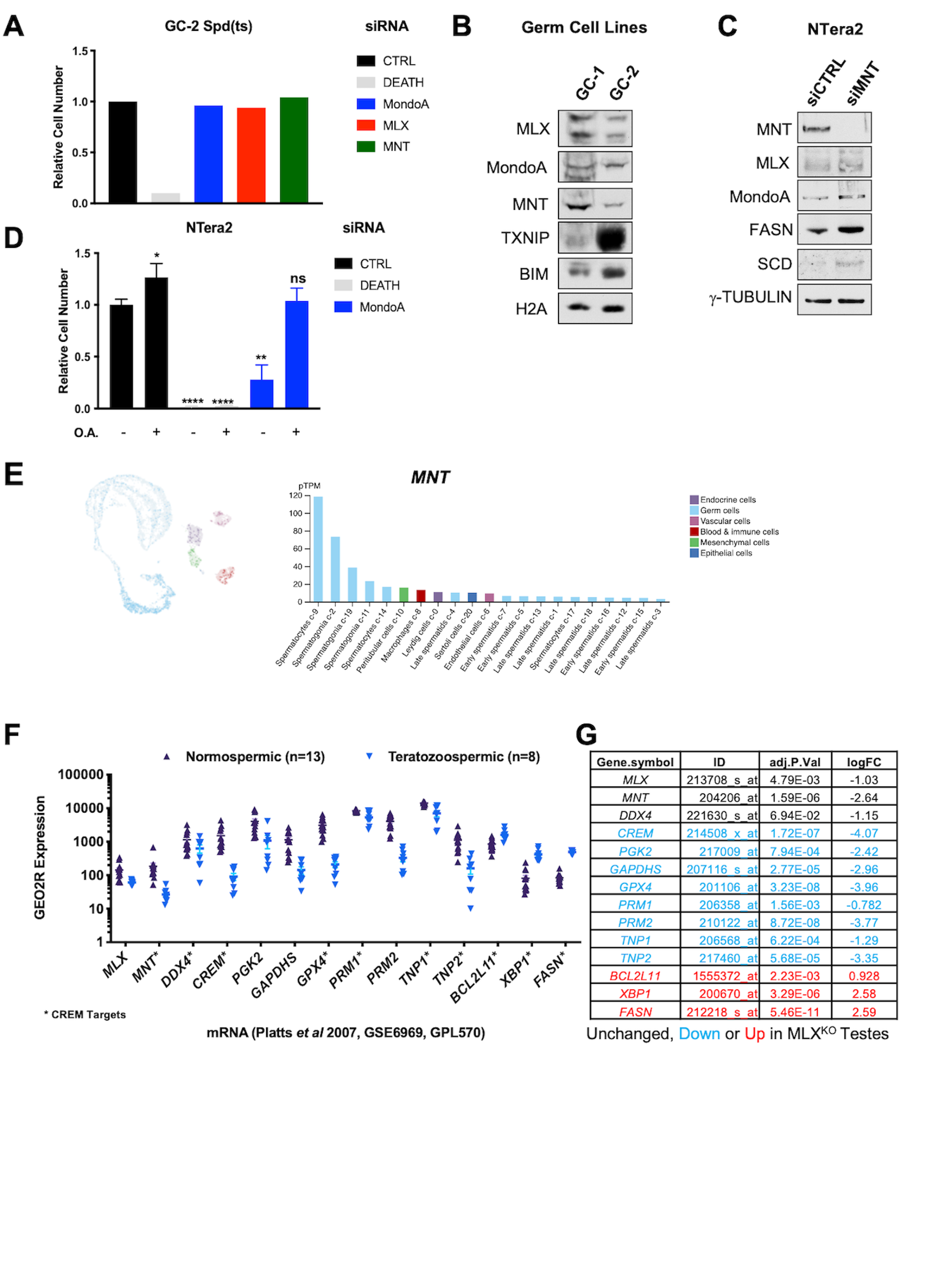

Supplement: S9 Fig — MLX and MNT regulate metabolism and survival of male GC lines. (A) Relative viable cell number of the GC-2 Spd(ts) cells after siRNA transfection with the indicated siRNA with siDEATH included as a control for siRNA transfection efficacy. (B) WB analysis of GC-1-Spg and GC-2 Spd(ts) cells probed for the indicated proteins. (C) WB analysis of NTera2 cells transfected with the indicated siRNA and probed for the indicated proteins. (D) Relative viable cell number of the NTera2 cells after siRNA transfection with the indicated siRNA with siDEATH included as a control for siRNA transfection efficacy. Cells were cultured in the presence or absence of 35 uM OA (N = 4 independent experiments, Shown is the mean +/− SEM), p-values shown from 1-way ANOVA with a Dunnett test compared with the Control (* p < 0.05, ** p < 0.01, *** p < 0.001, *** p < 0.0001). (E) Analysis of MNT expression from the Human Protein Atlas Single-cell RNA-seq dataset [23] Image credit: Human Protein Atlas. Image available from v20.1.proteinatlas.org (http://www.proteinatlas.org). (F, G) GEO2R analysis of GSE6969 from a published dataset of fertile (normospermic) versus infertile (teratozoospermic) men. LogFC and p-values are shown on the table to the right. The underlying data for S9A, S9D, and S9F Fig can be found in S1 Data. GC, germ cell; MLX, MAX-Like protein X; OA, oleic acid; RNA-seq, RNA sequencing; siRNA, small interfering RNA; WB, western blot. (TIF) [file pbio.3001085.s009.tif]
