# Supplementary material for: The glucose-sensing transcription factor MLX balances metabolism and stress to suppress apoptosis and maintain spermatogenesis
Source: PLoS Biol. 2021 Oct 20;19(10):e3001085. doi: 10.1371/journal.pbio.3001085 (PMC8528285; doi:10.1371/journal.pbio.3001085)

S1_RAW_IMAGES

Rescanned full lane images of original gels and film images used to make all main and supplemental figures. Some western blots have been cut and/or reprobed. Relevant bands scanned in the original are marked in red boxes. These images were not cropped to make the figures, as the original scans were carried out as far back as 2014, and the scanned area was limited to the actual image in those figures. These are rescans of those original films.

FIGURE 1A

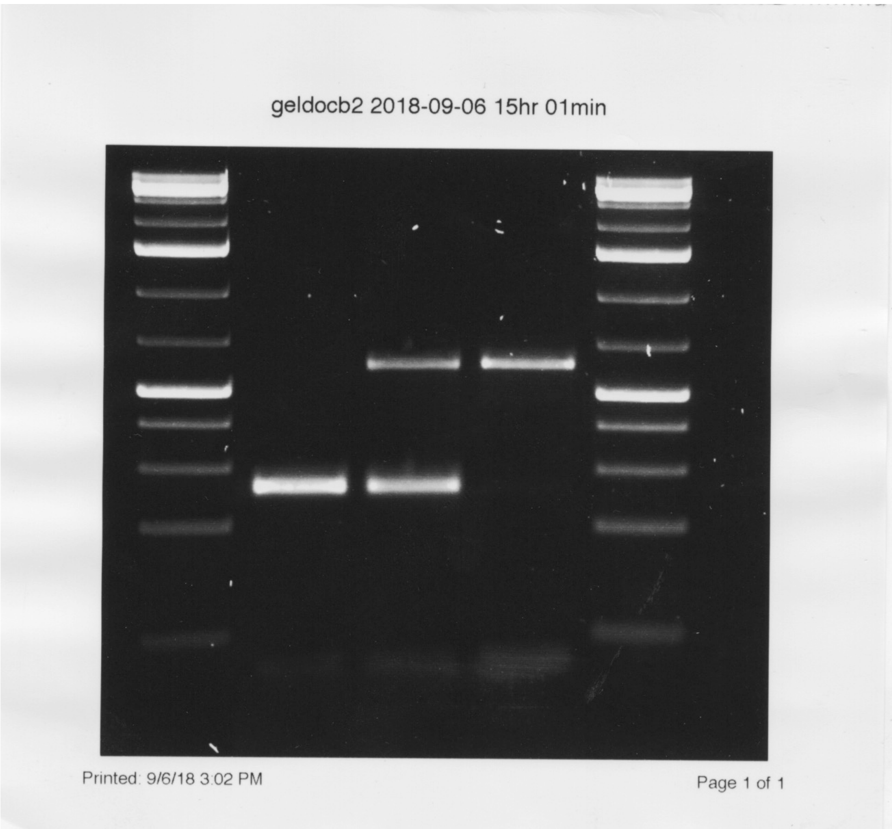

FIGURE S5B

MLX

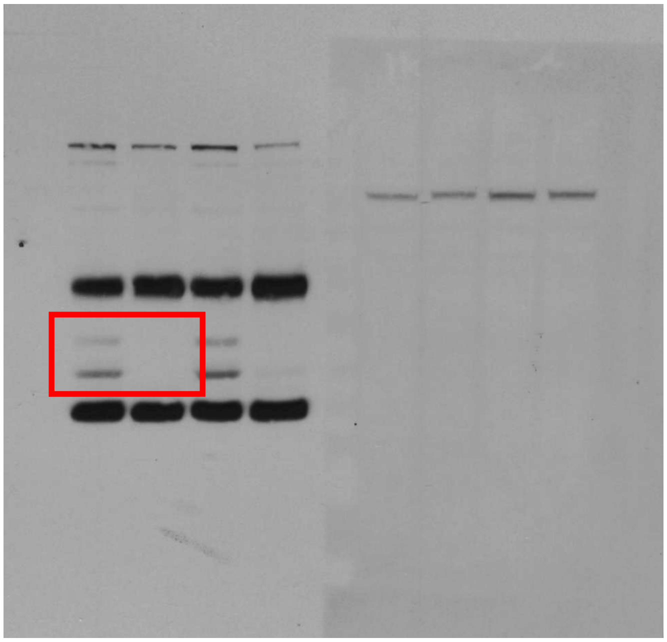

MondoA

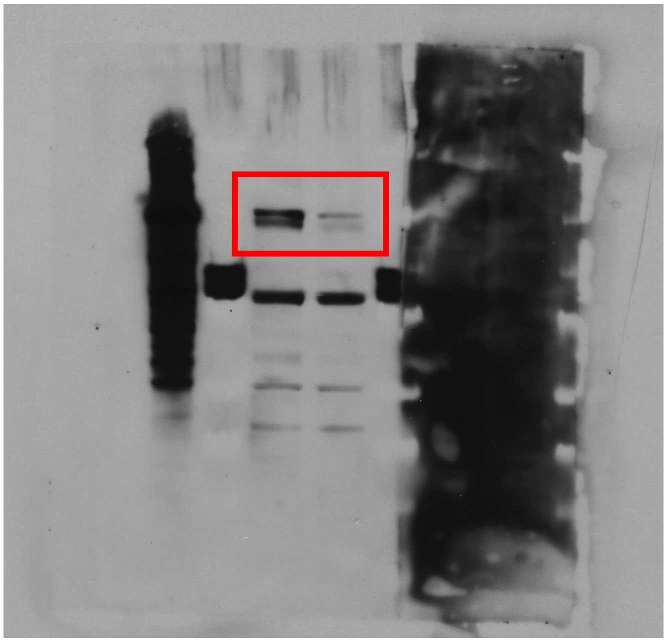

ChREBP

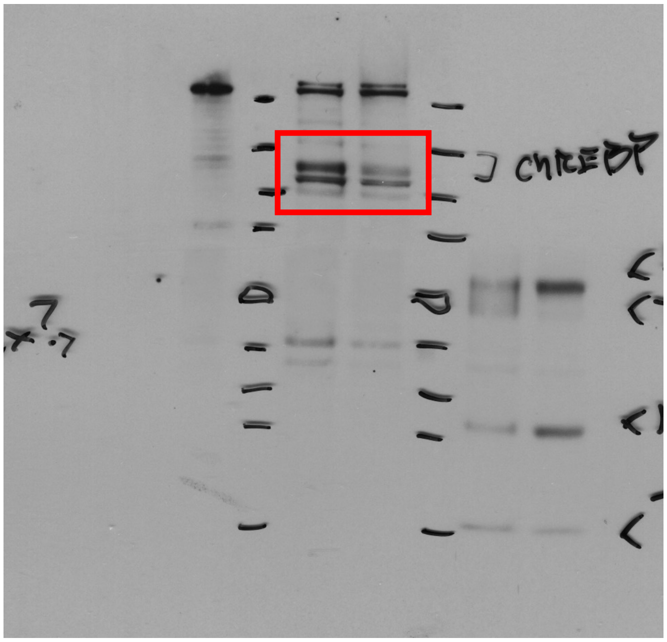

TXNIP

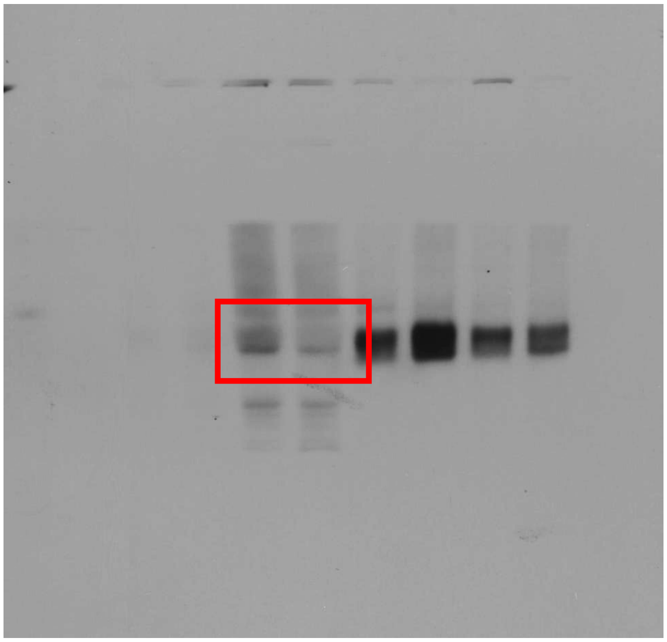

FIGURE S5B

EOMES

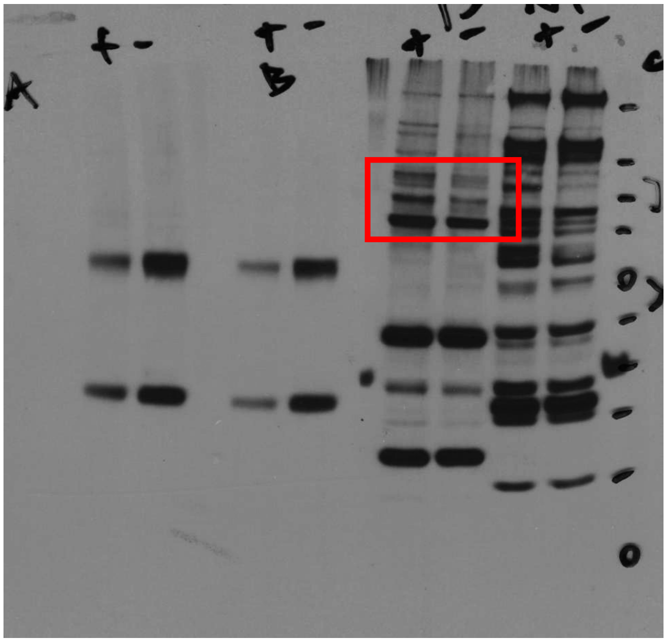

FAS

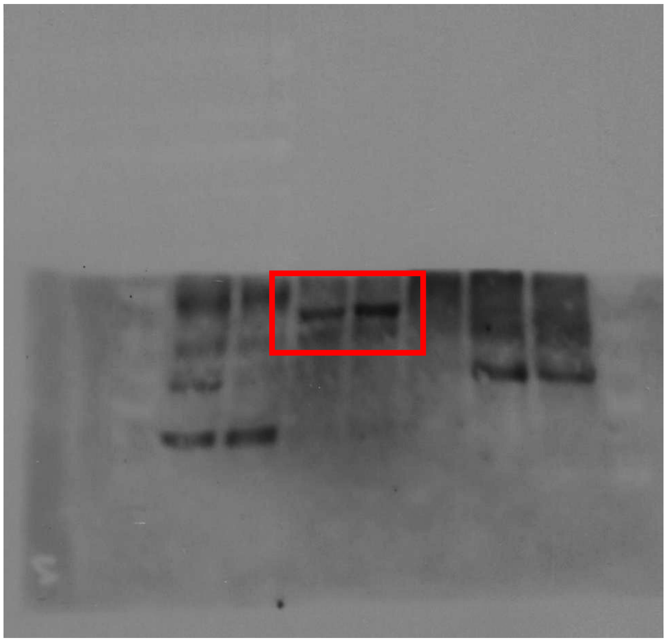

TUBULIN and ACTIN

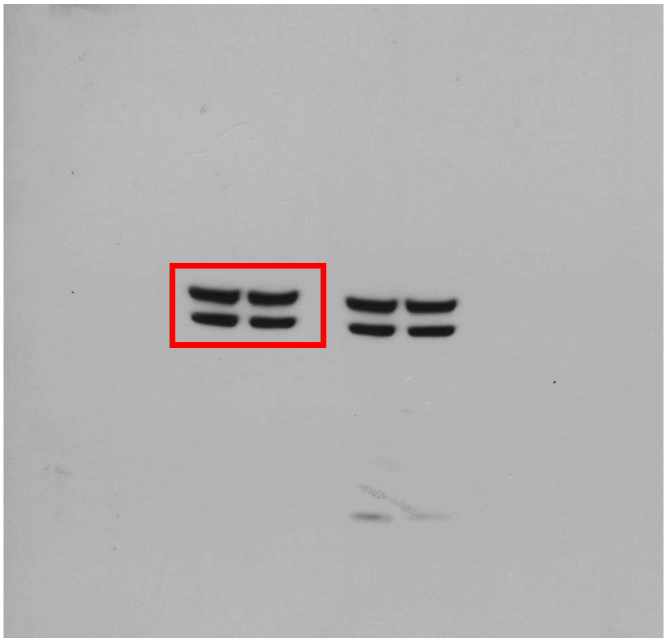

FIGURE S5C

IMMUNOGLOBULIN

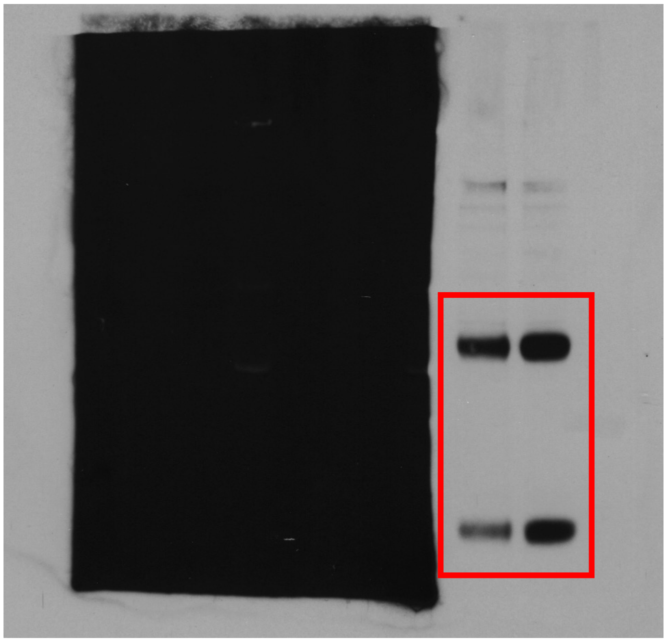

TUBULIN and ACTIN

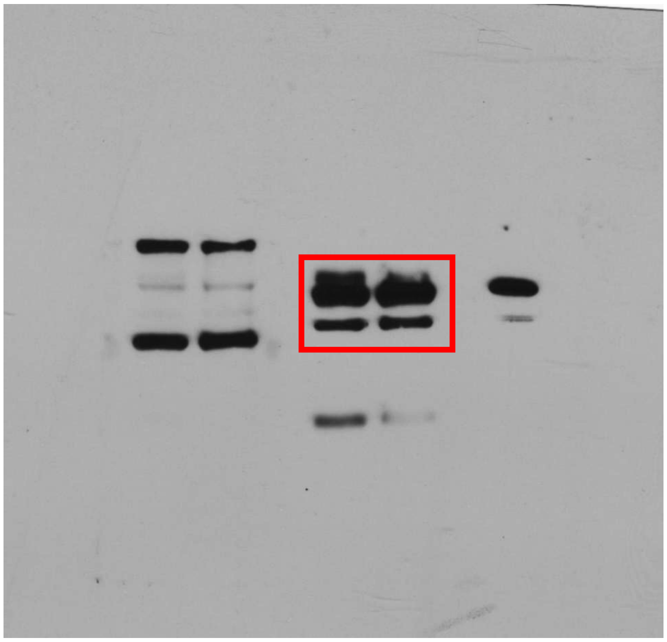

FIGURE 6A

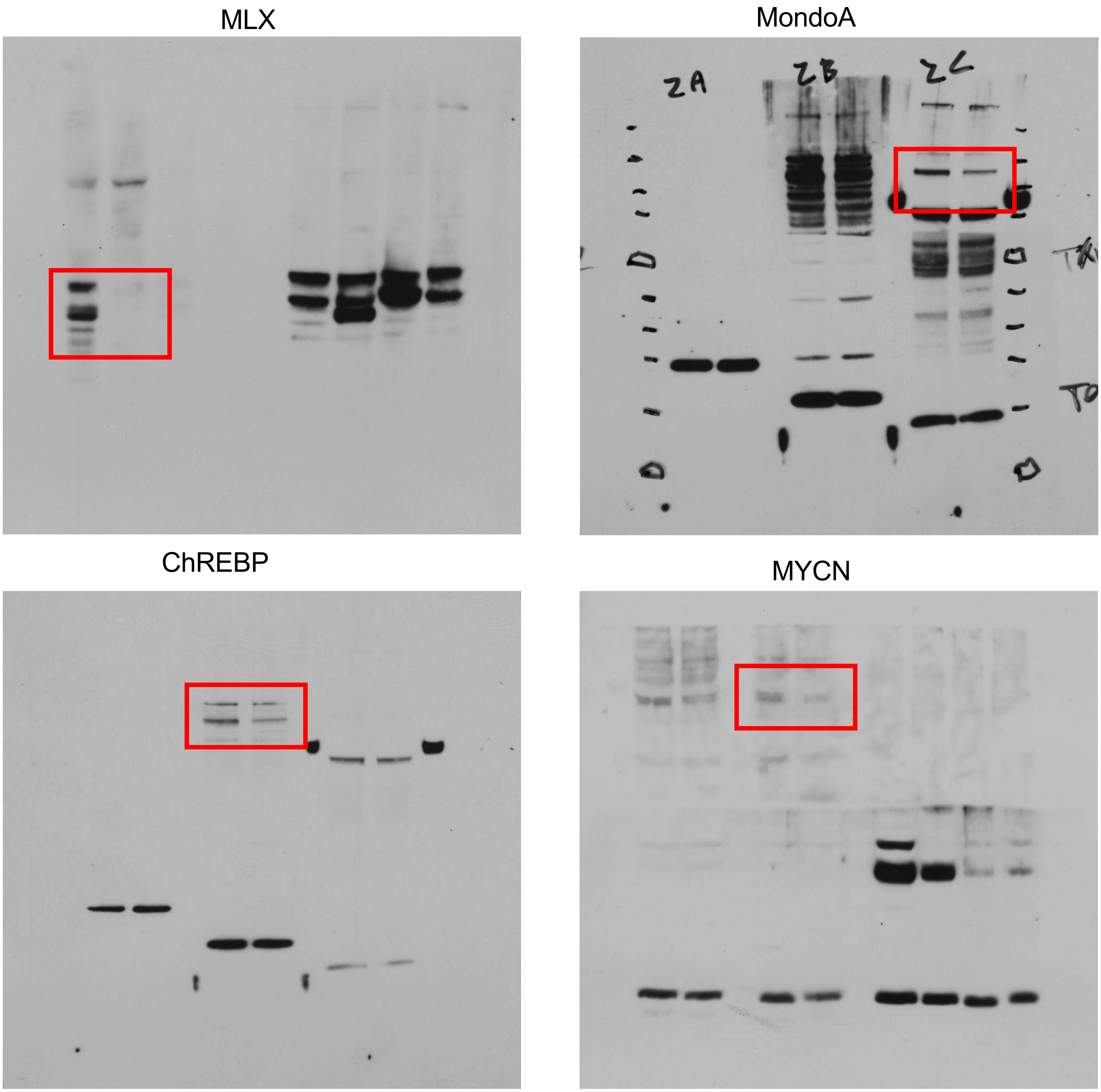

FIGURE 6A

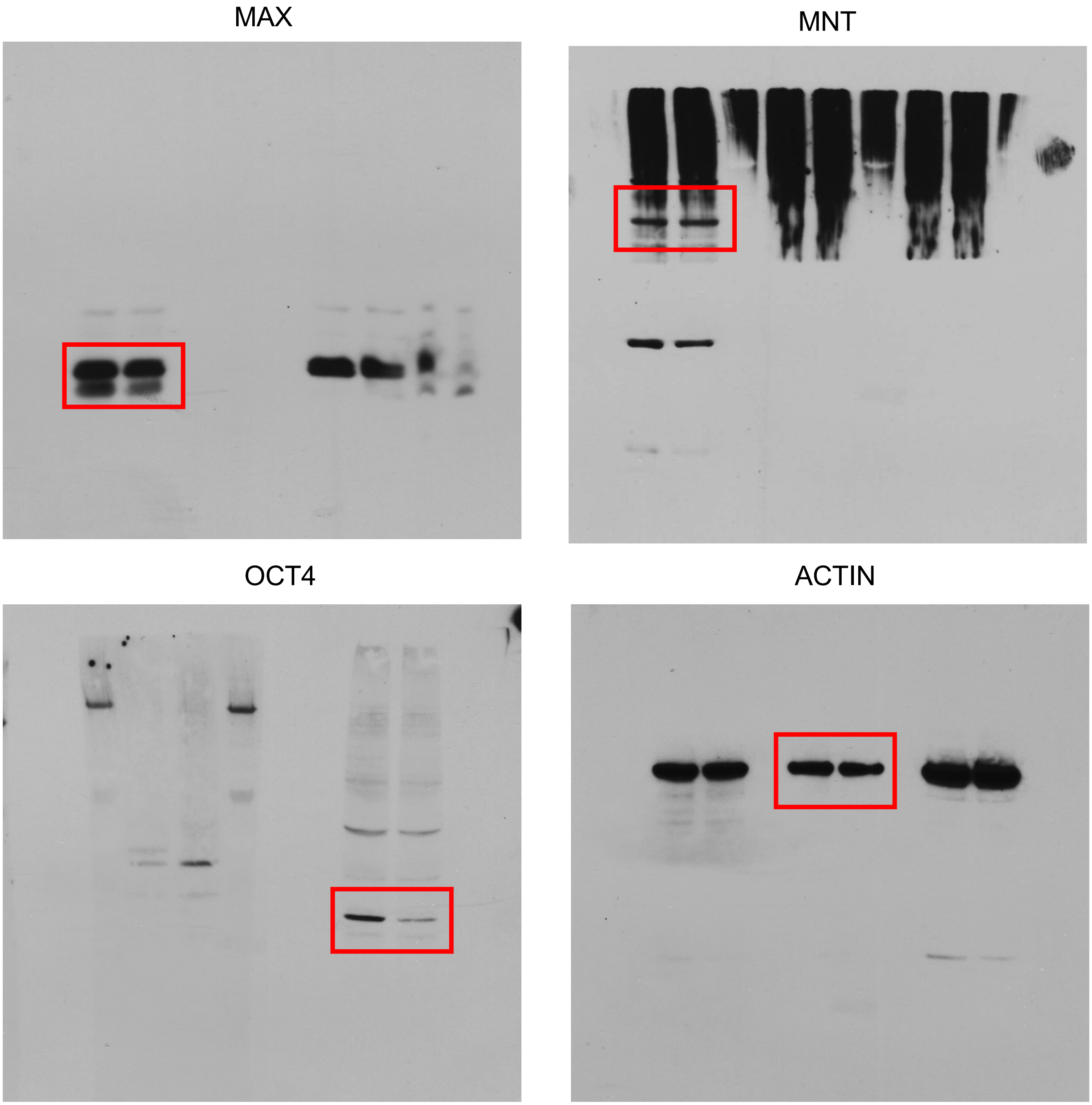

FIGURE 6B (Shares some panels with S6C)

MLX

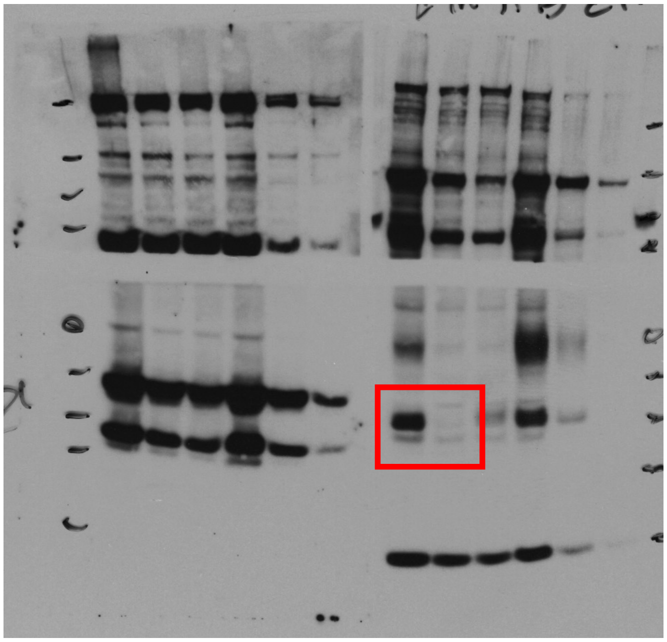

MondoA

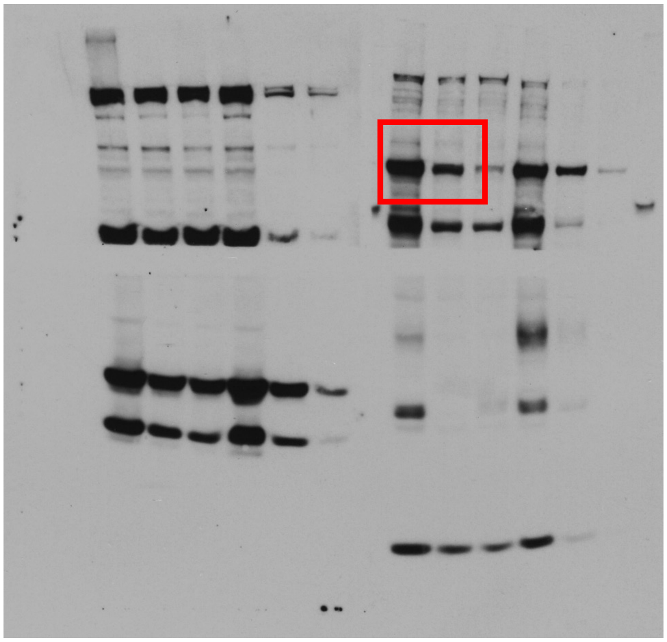

ChREBP

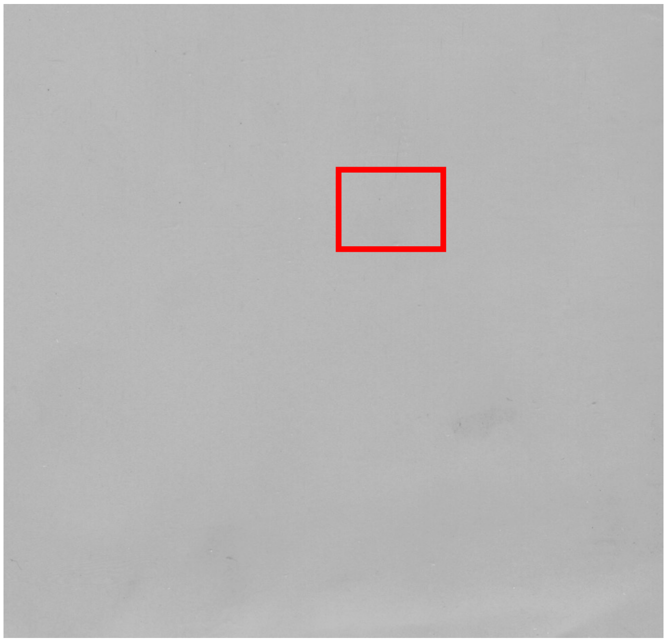

MYCN

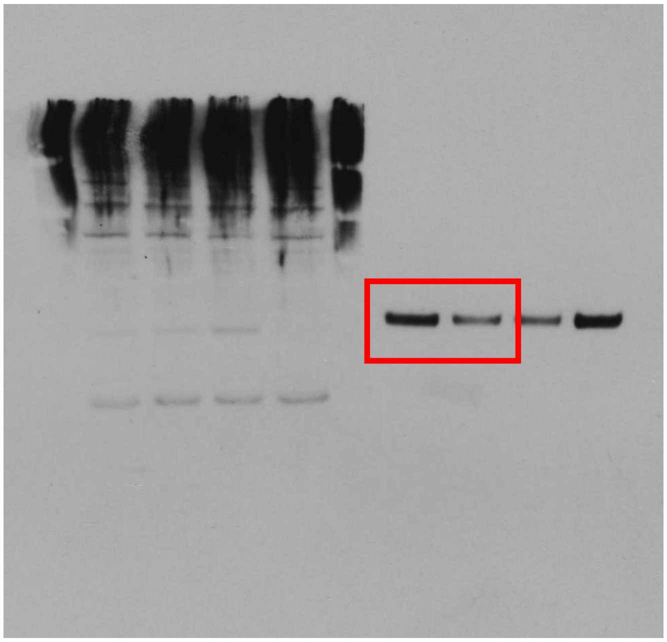

FIGURE 6B (Shares some panels with S6C)

MAX

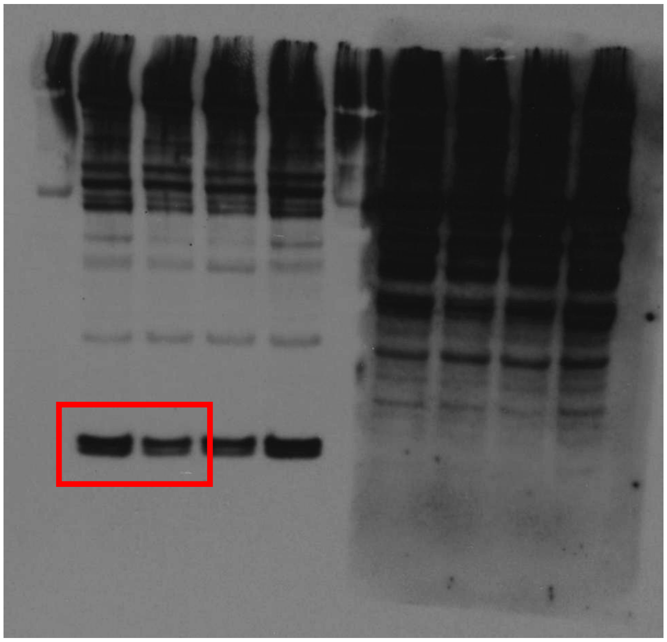

MNT

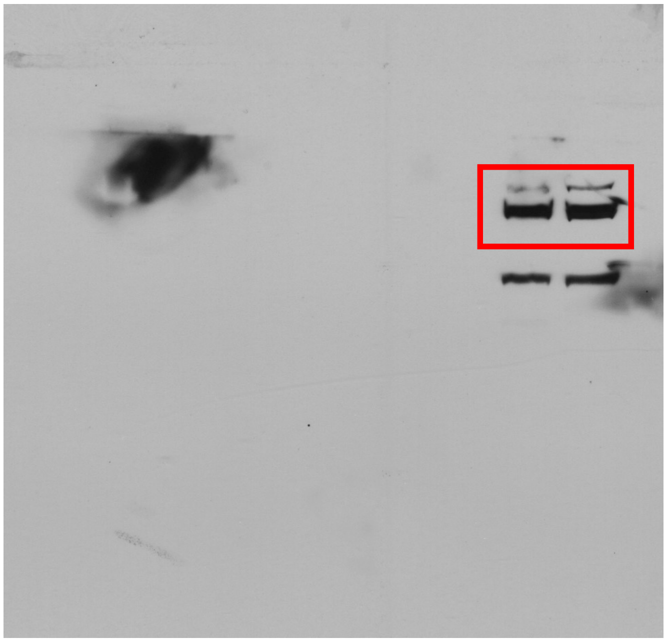

OCT4

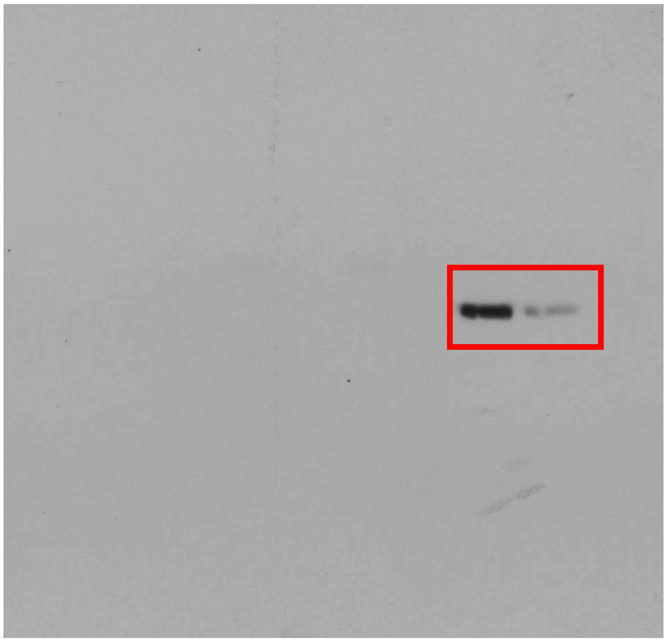

ACTIN

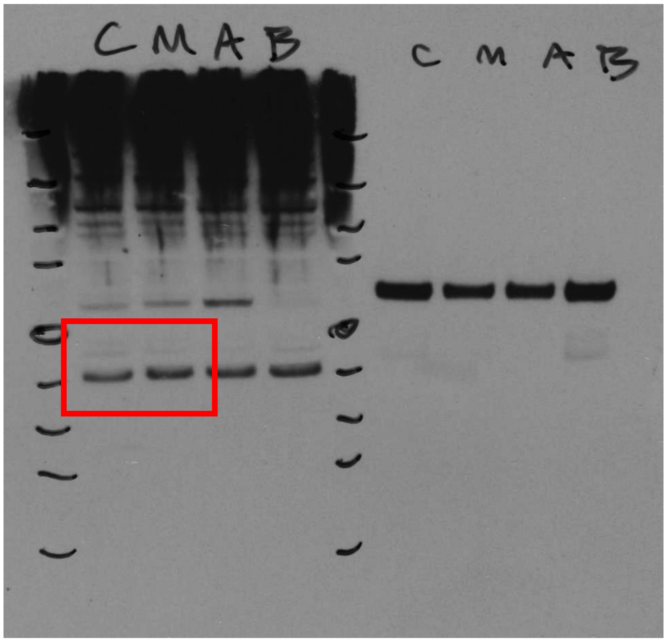

FIGURE 6C

DDX4

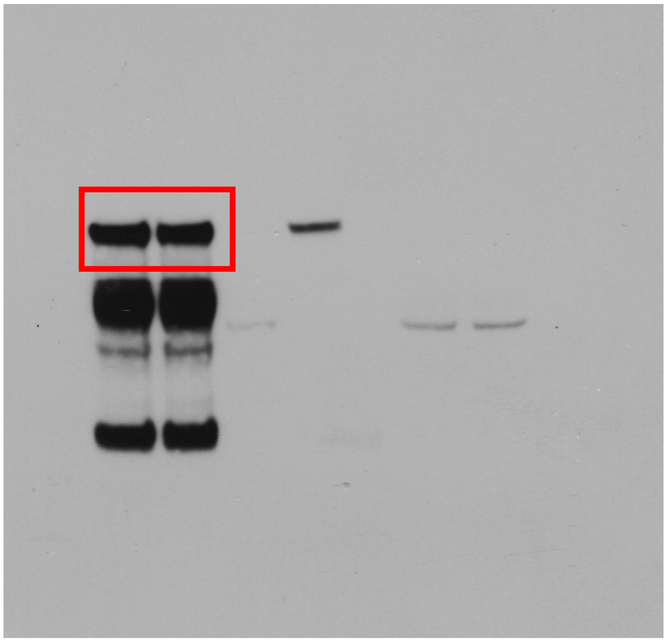

PGK2

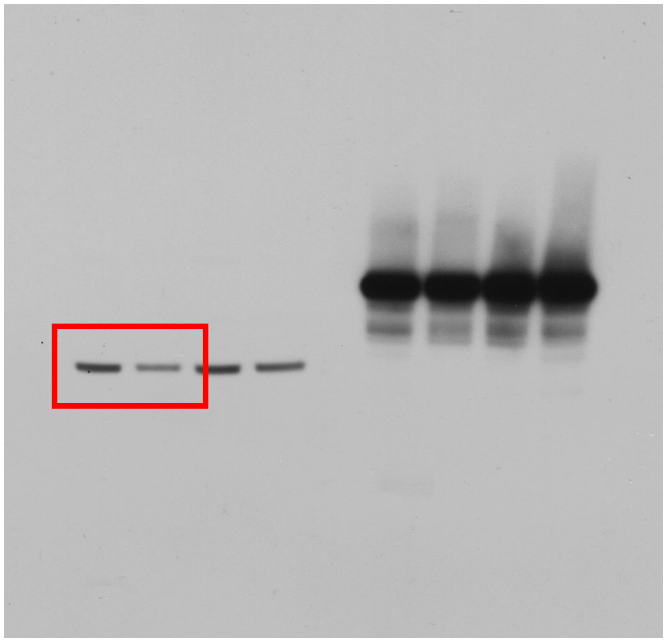

TXNIP

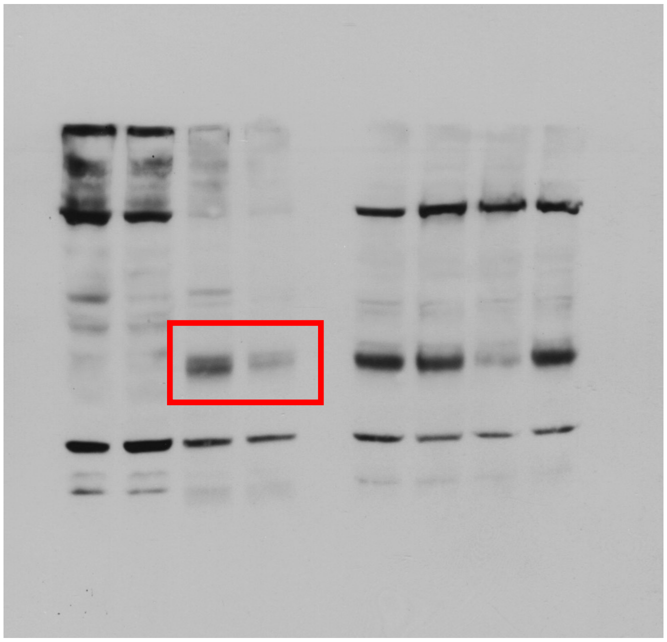

CPT1A

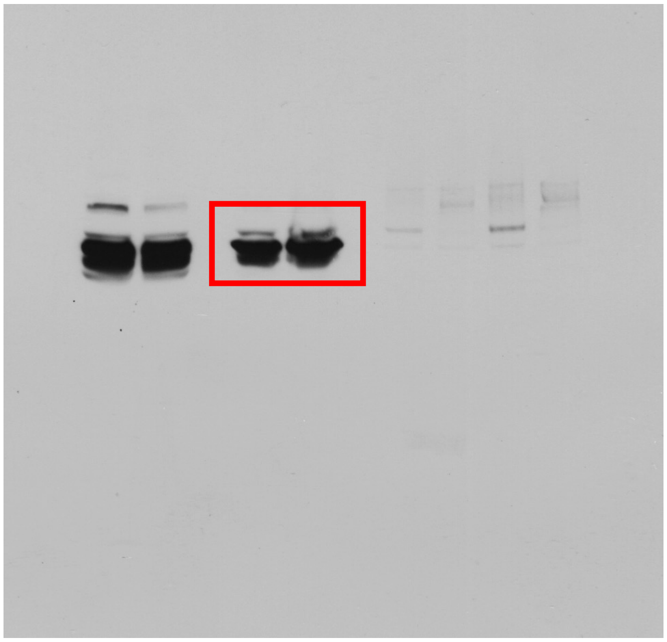

FIGURE 6C

IGFBP3

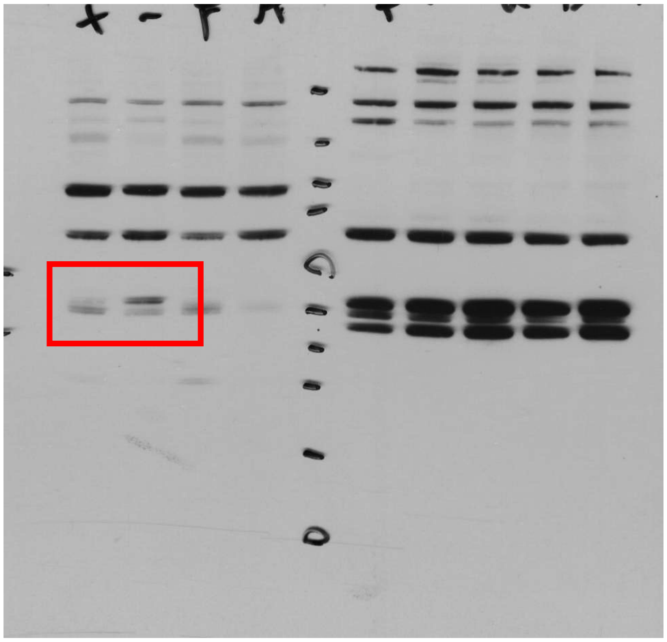

FAS

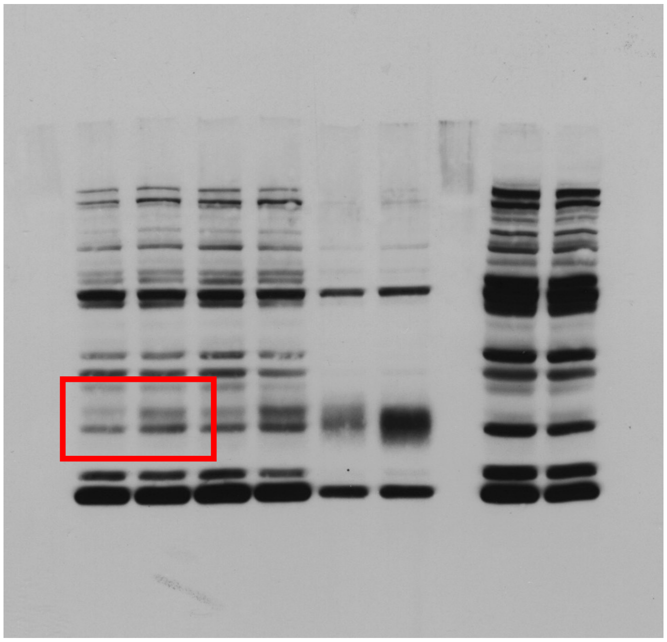

γH2AX

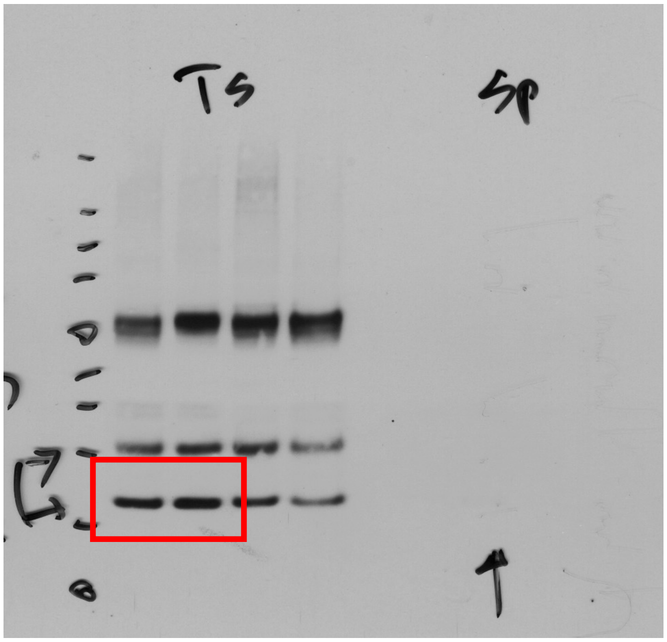

PARP and BIM

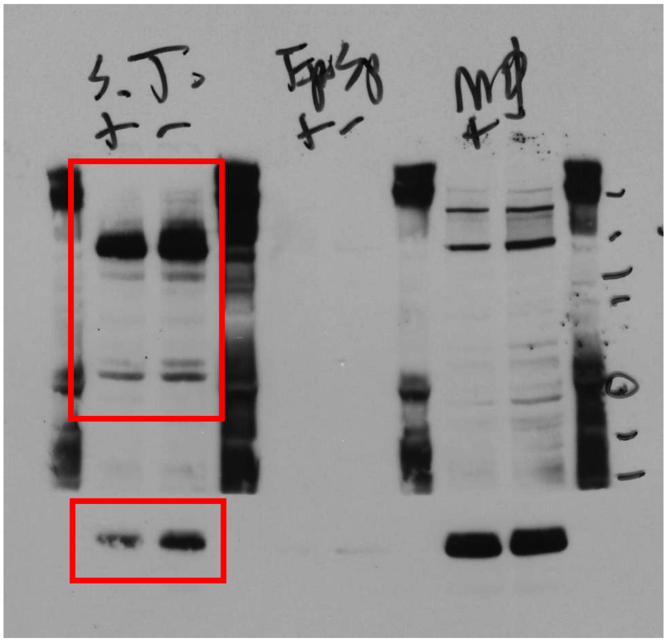

FIGURE 6C

ACTIN

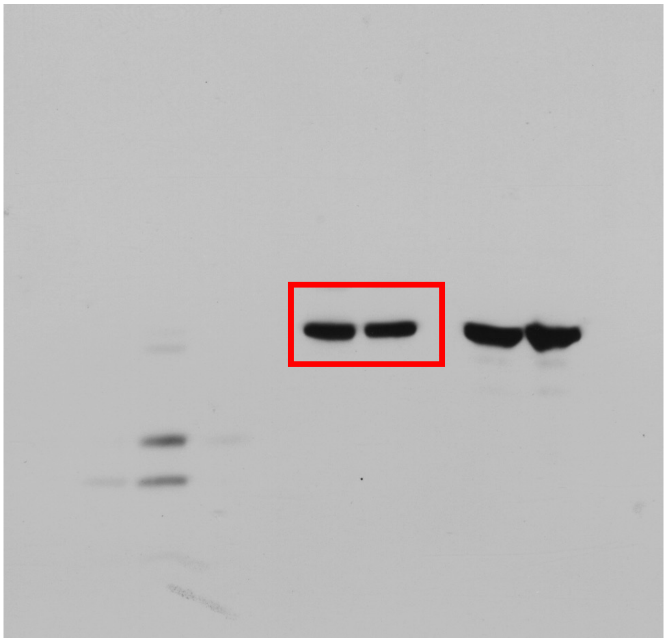

FIGURE 6D

DDX4

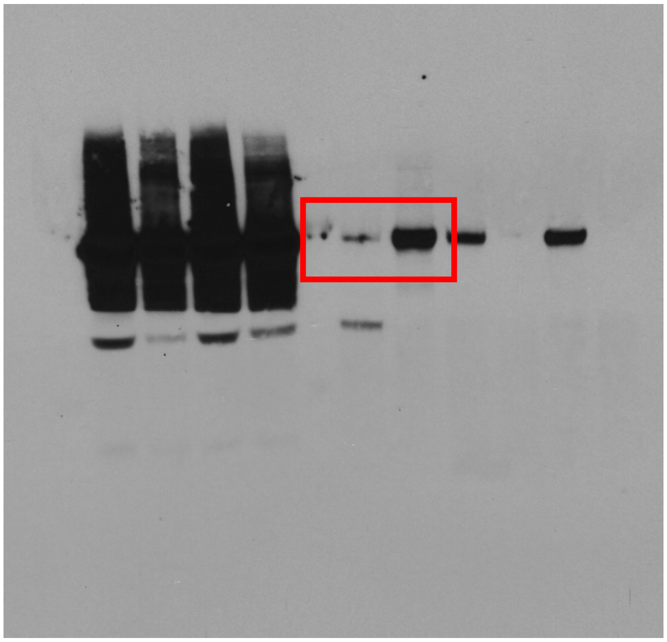

PGK2

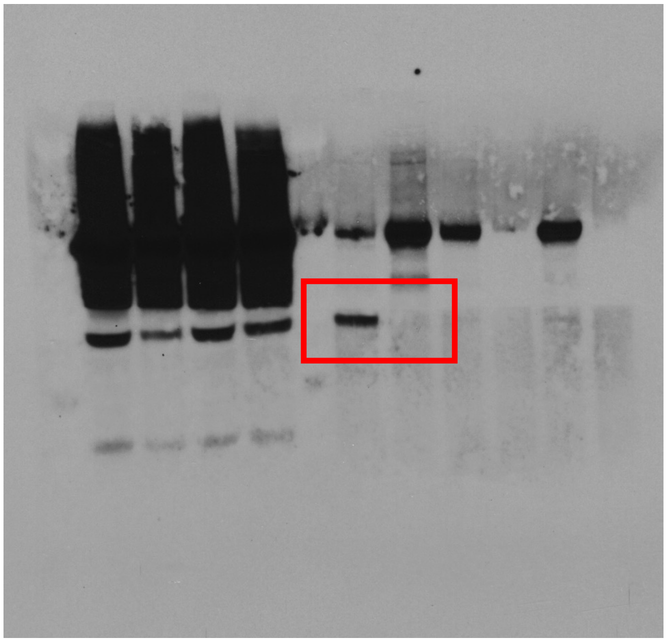

TXNIP

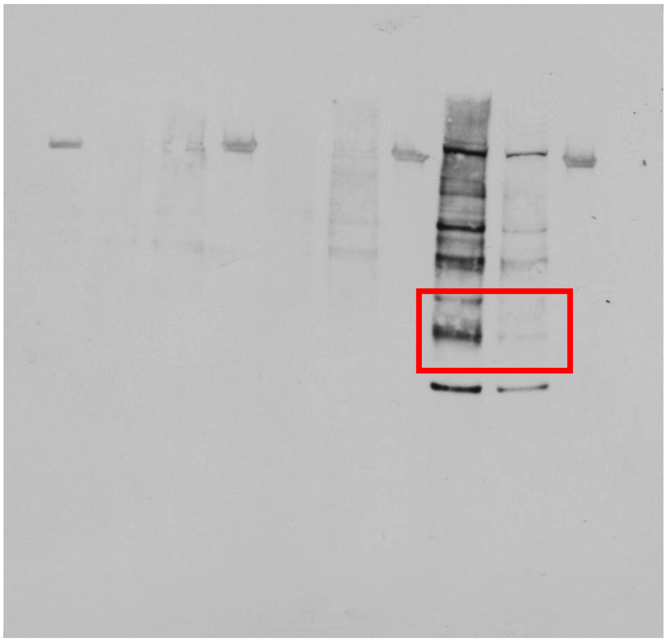

CPT1A

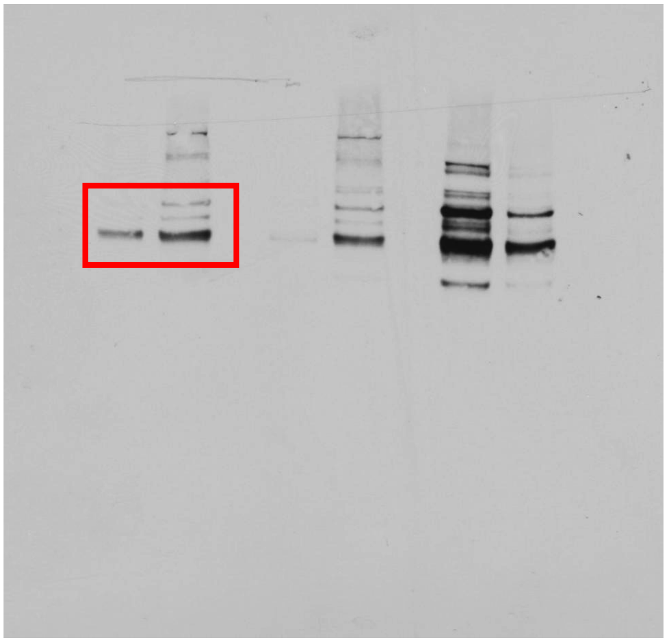

FIGURE 6D

IGFBP3

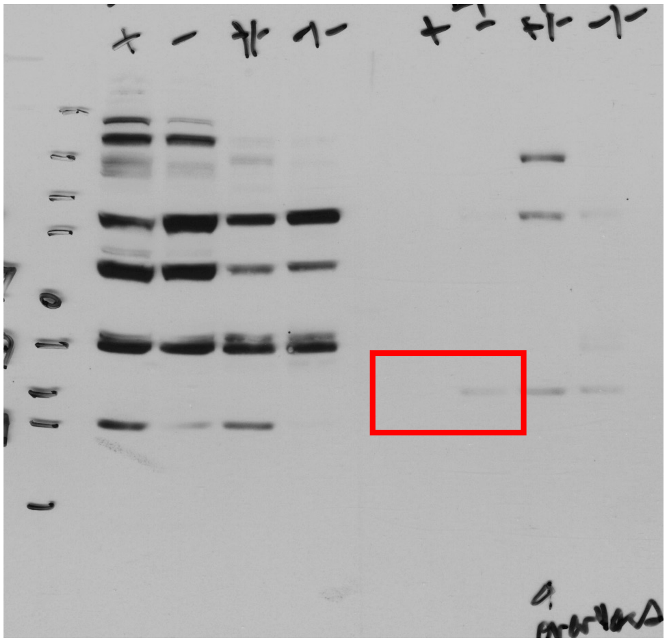

FAS

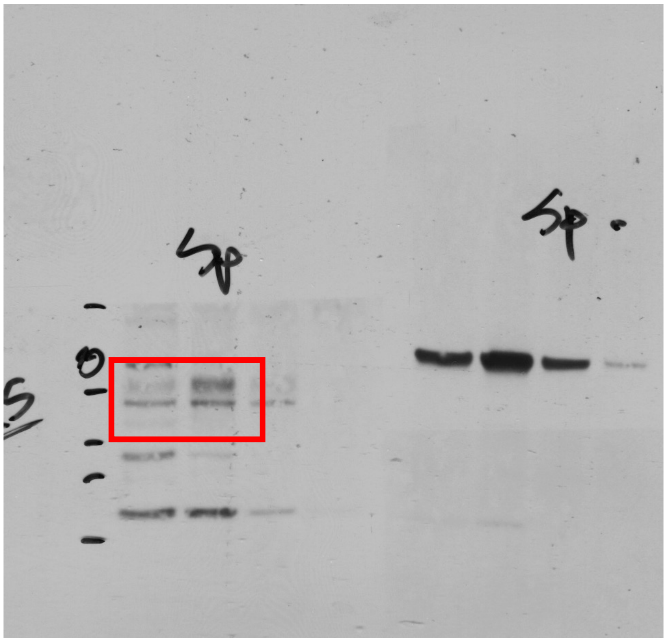

γH2AX

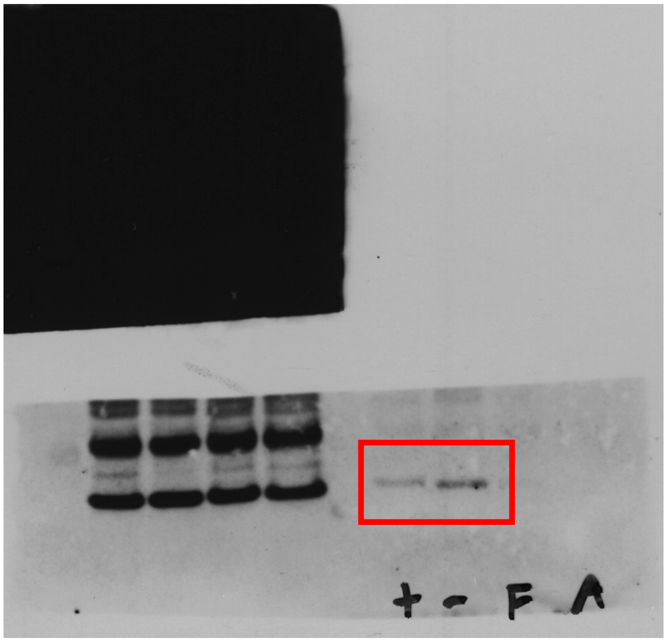

PARP and BIM

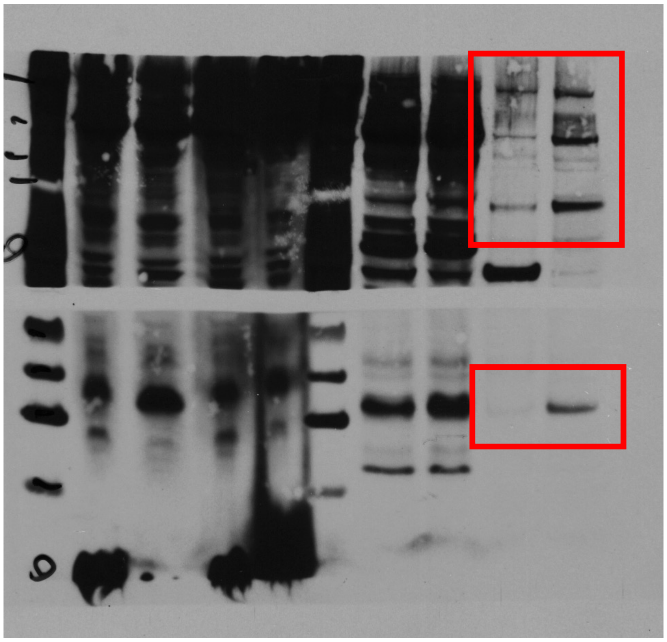

FIGURE 6D

ACTIN

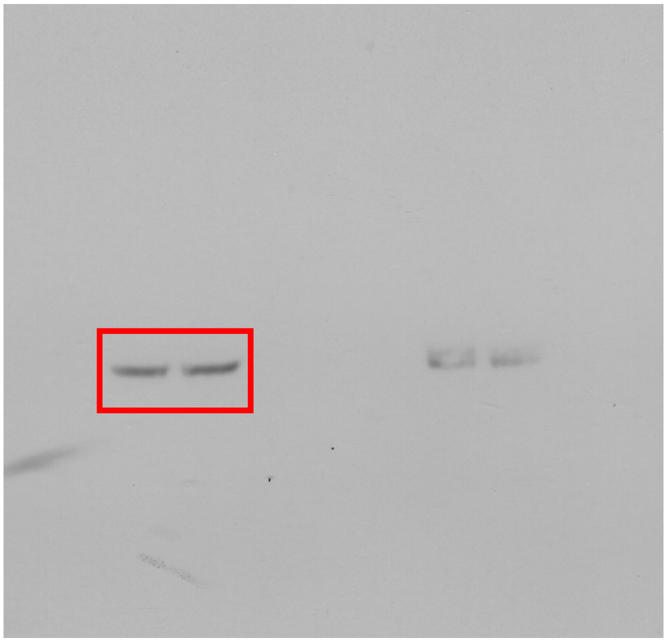

FIGURE S6A

MLX

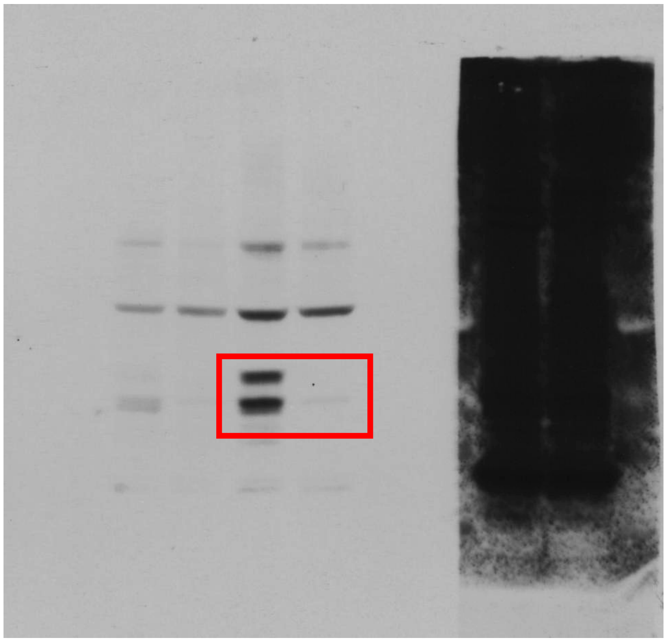

MondoA

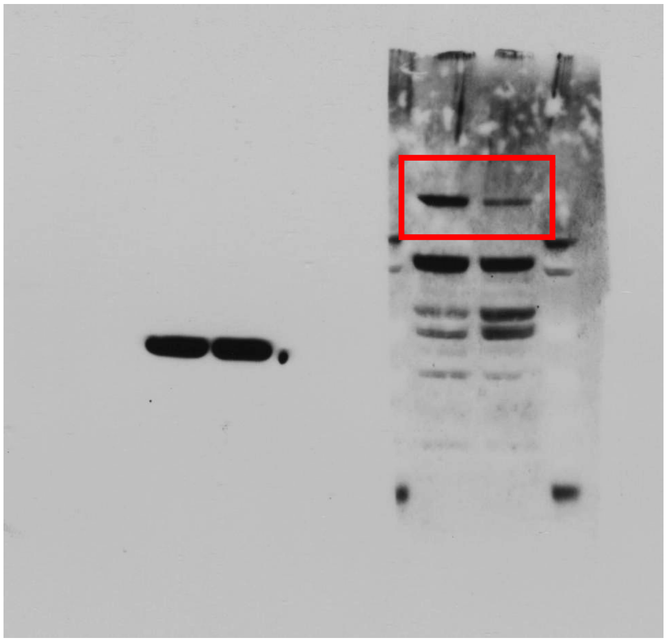

ChREBP

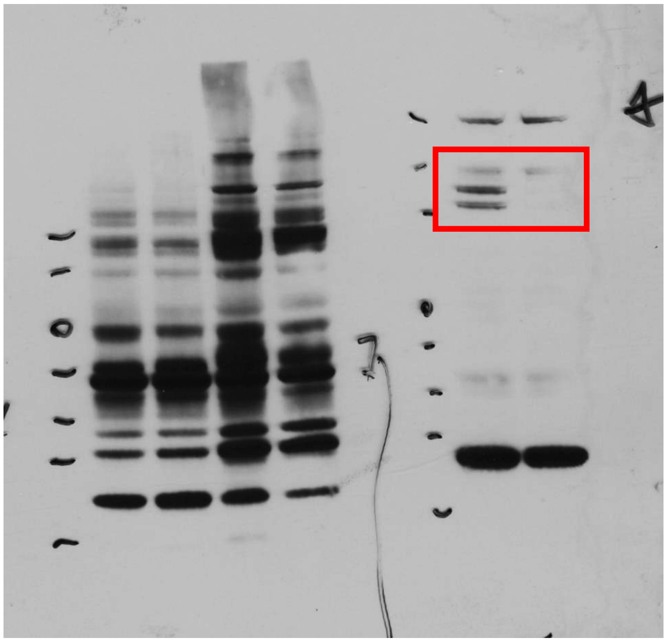

TXNIP

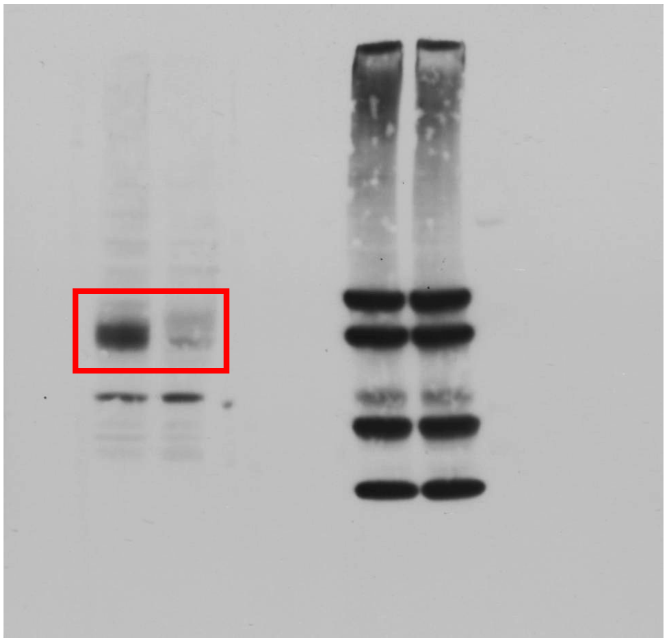

FIGURE S6A

CPT1A

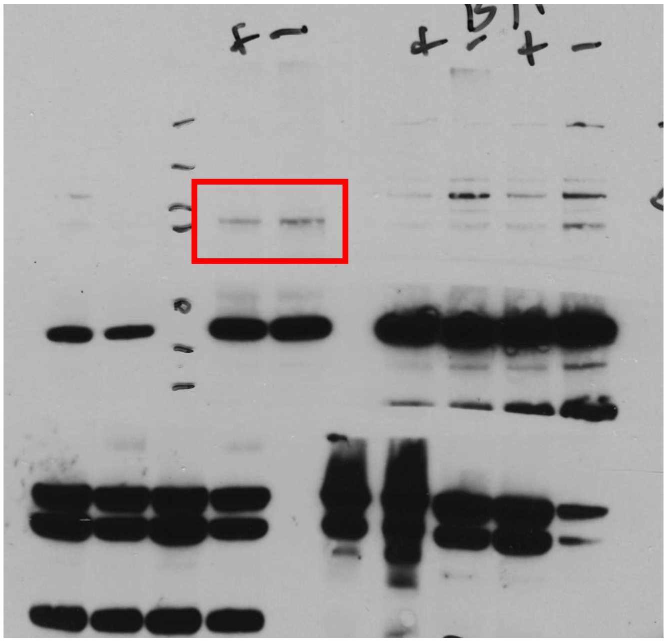

IGFBP3

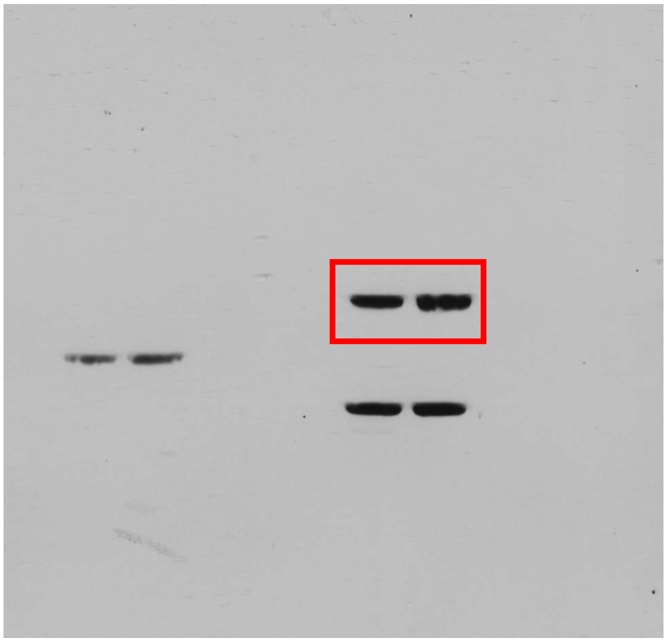

FAS

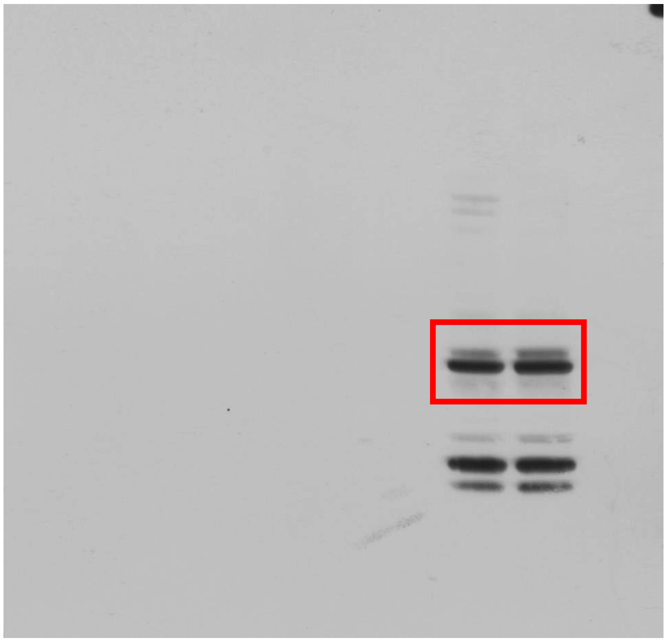

ACTIN

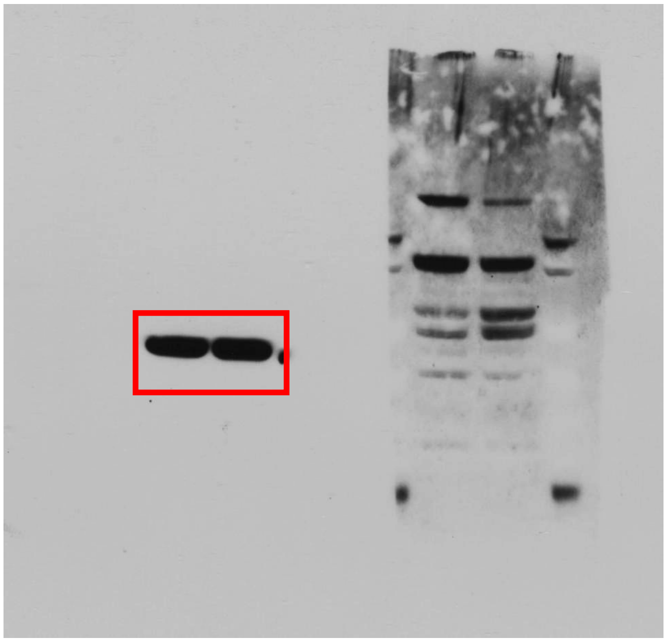

FIGURE S6B

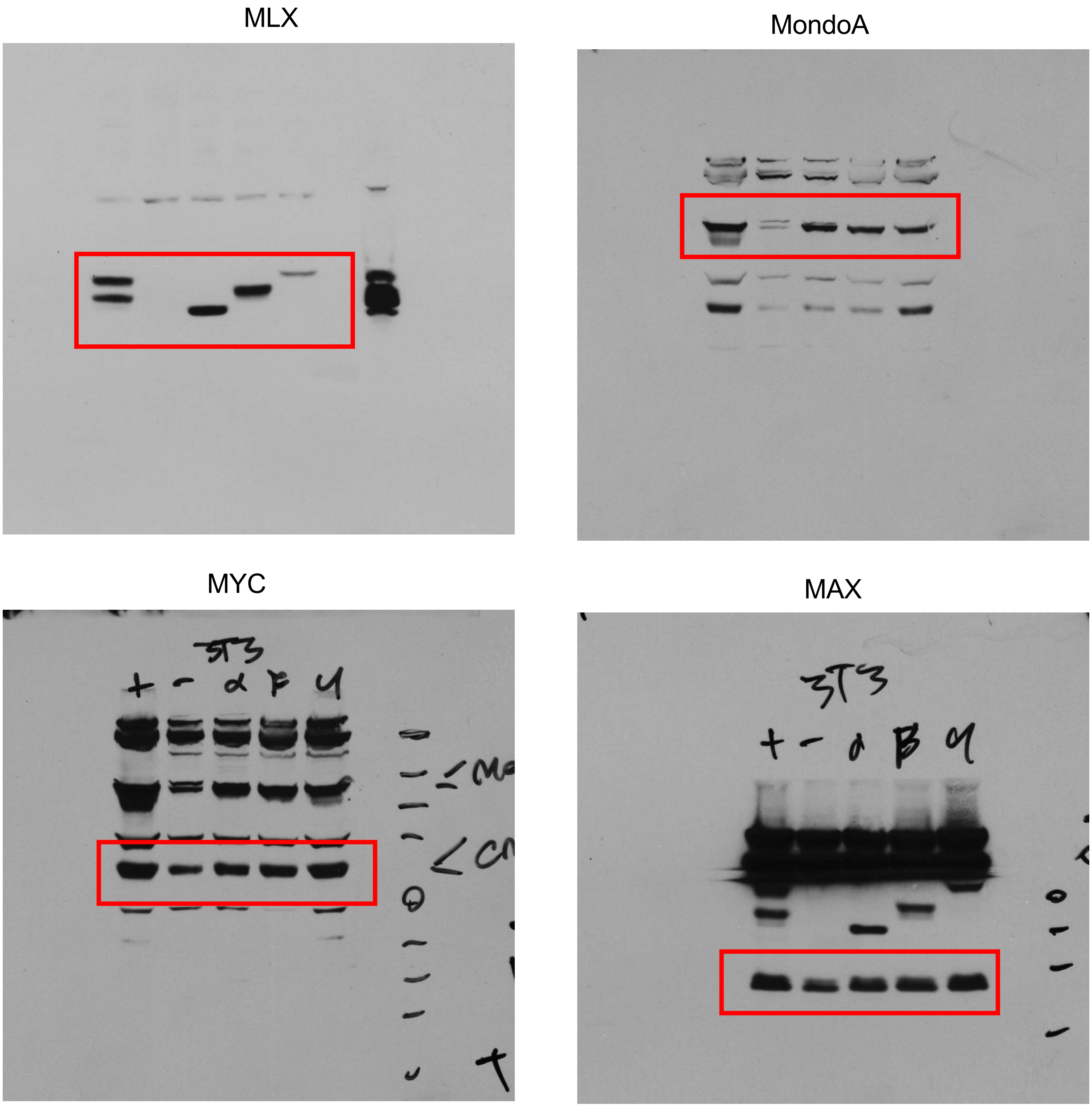

FIGURE S6B

FASN

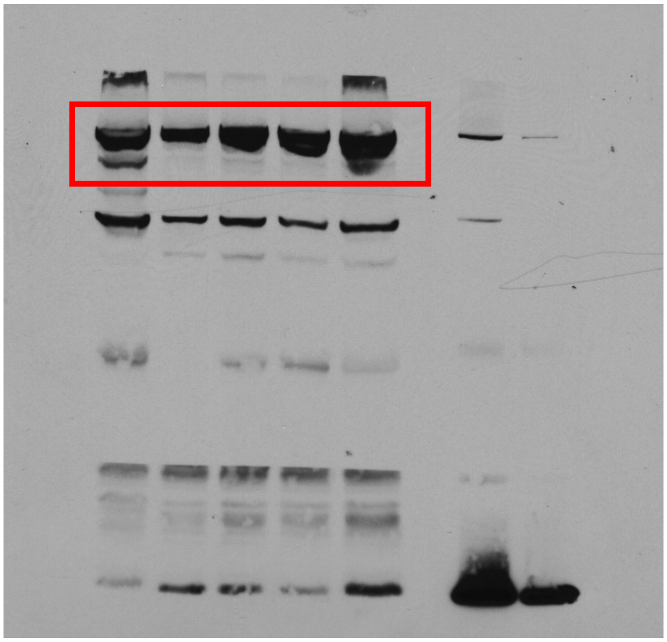

TXNIP

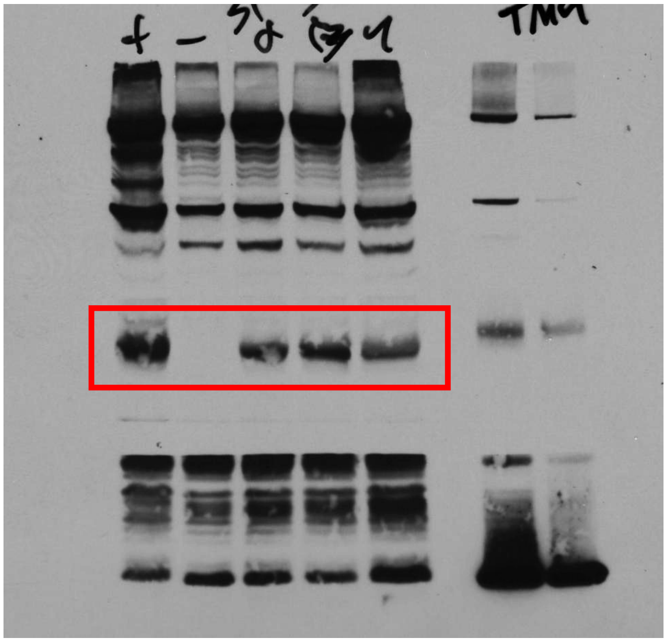

TOMM20

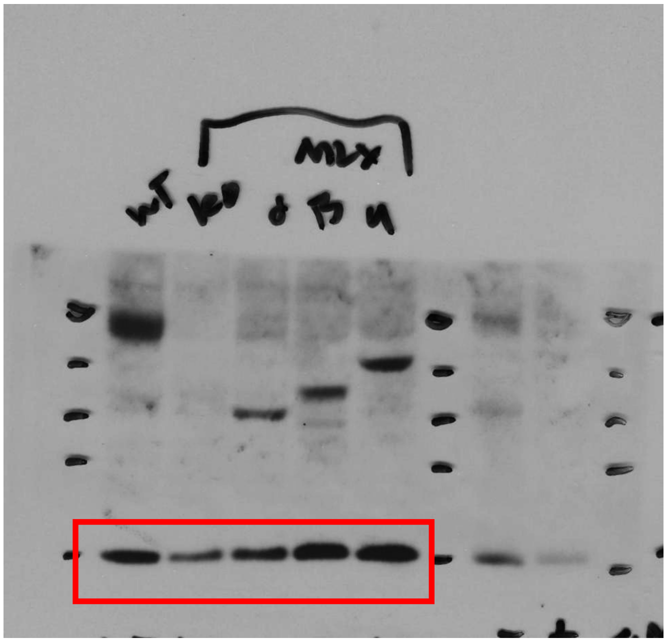

TUBULIN and FAS

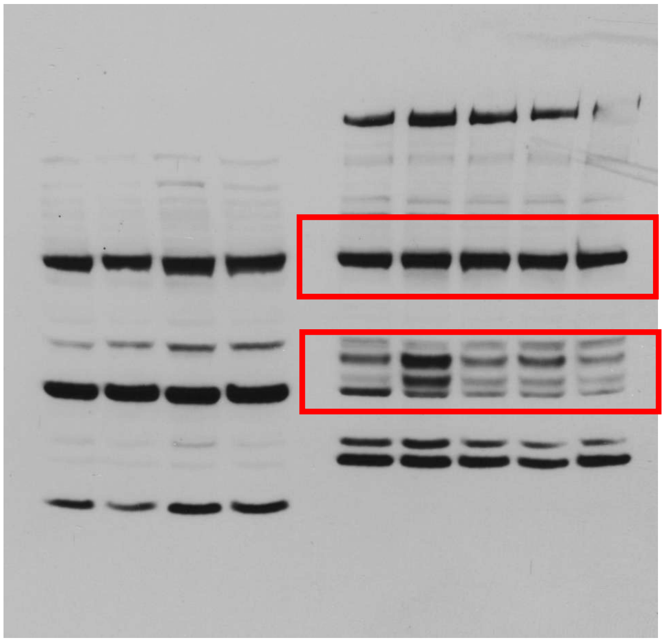

FIGURE S6C (A subset of panels are shared with Figure 6B)

TXNIP and MLX

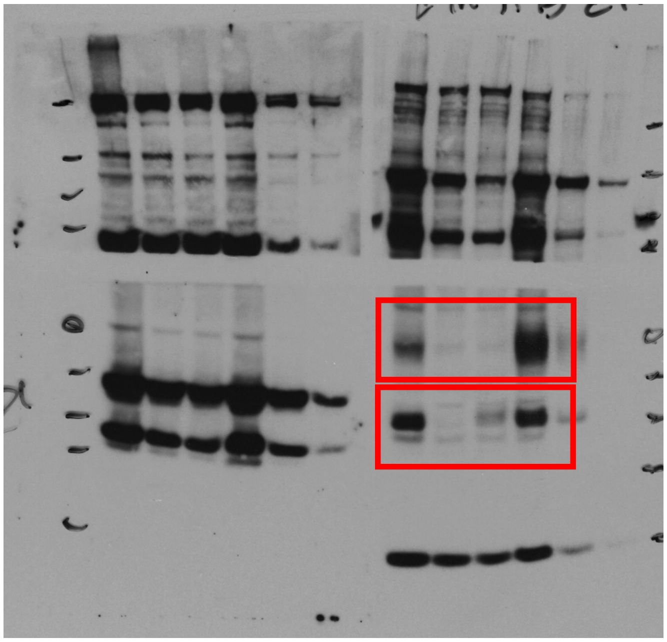

MondoA and TOMM20

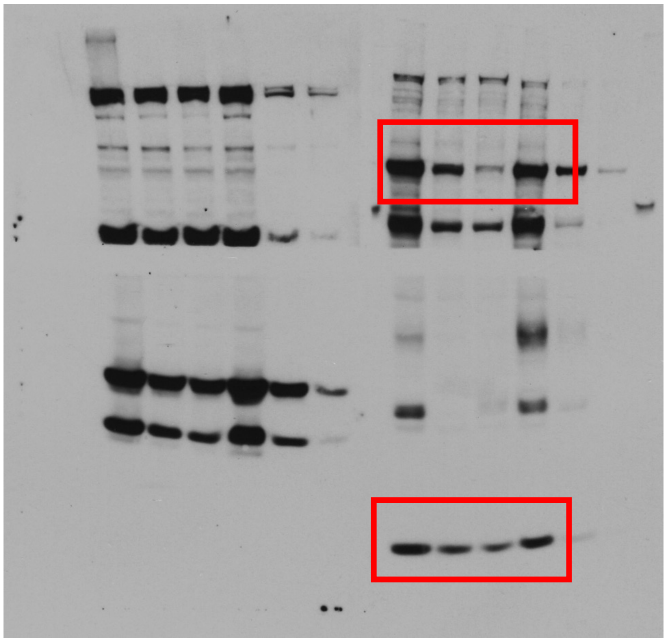

ChREBP

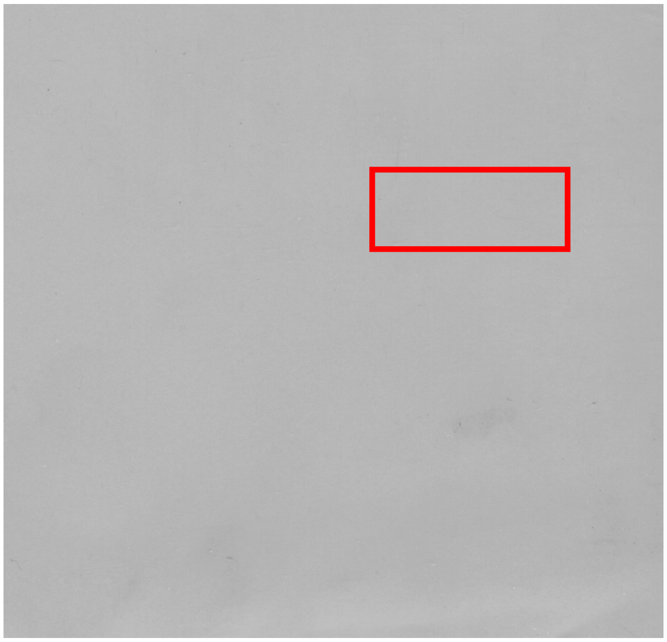

SCD

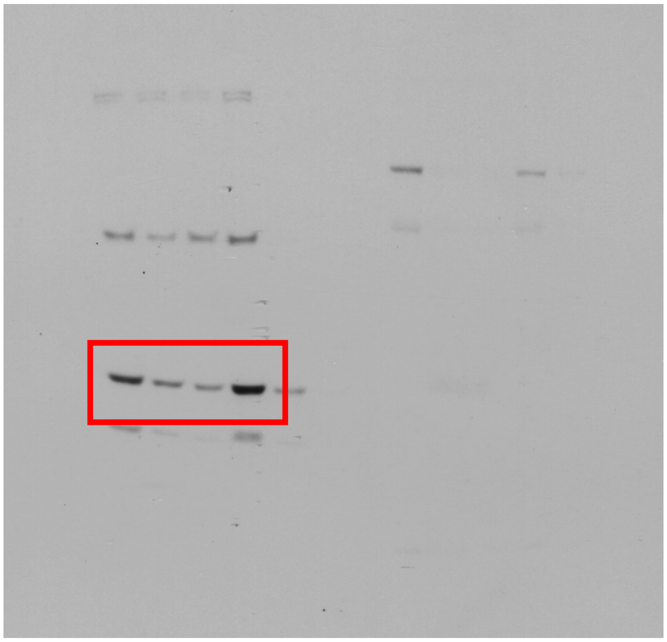

FIGURE S6C (A subset of panels are shared with Figure 6B)

MYCN

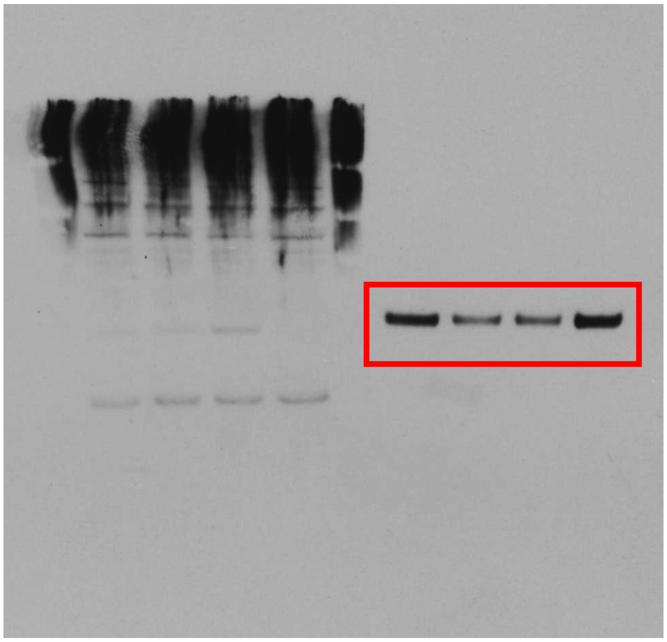

MAX

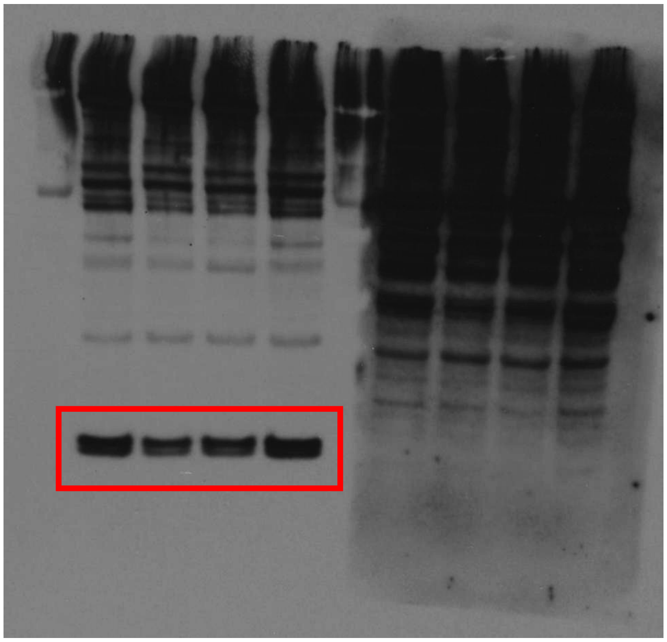

ACTIN

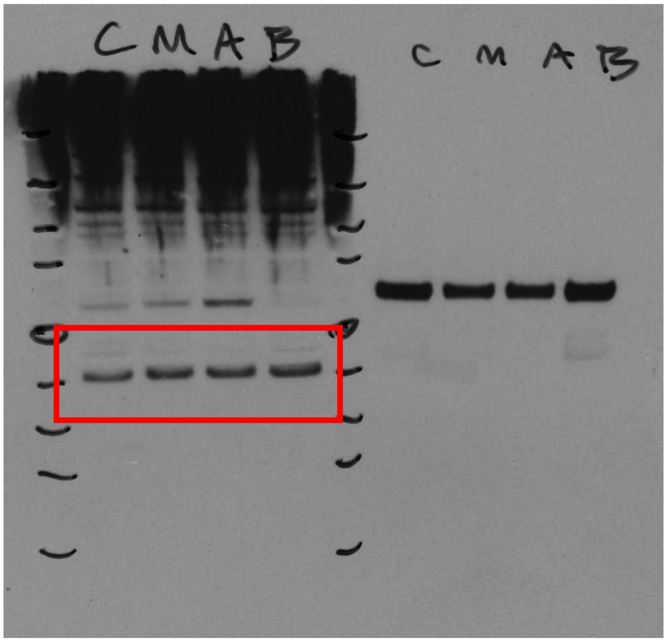

FIGURE 8E

MLX(MLX^{KO})

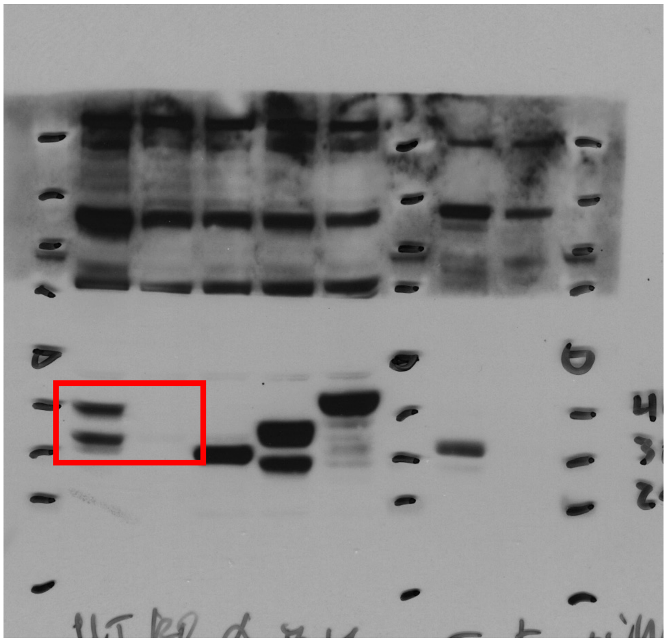

MLX(MNT^{KO})

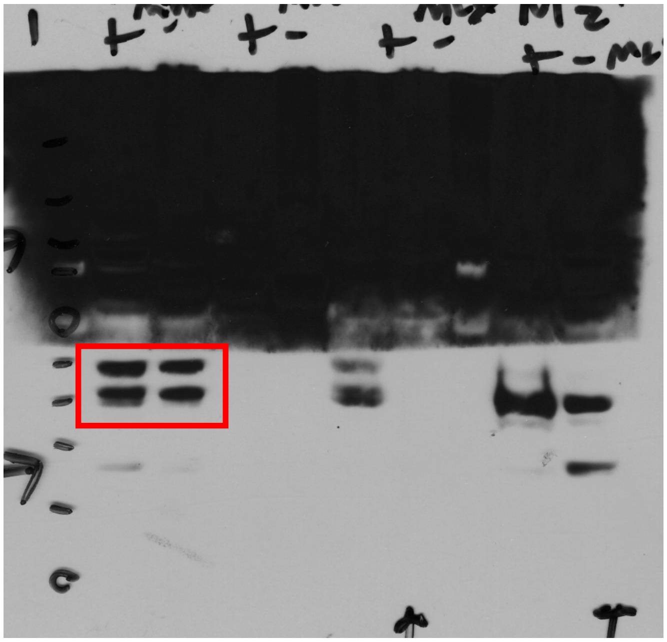

MondoA(MLX^{KO})

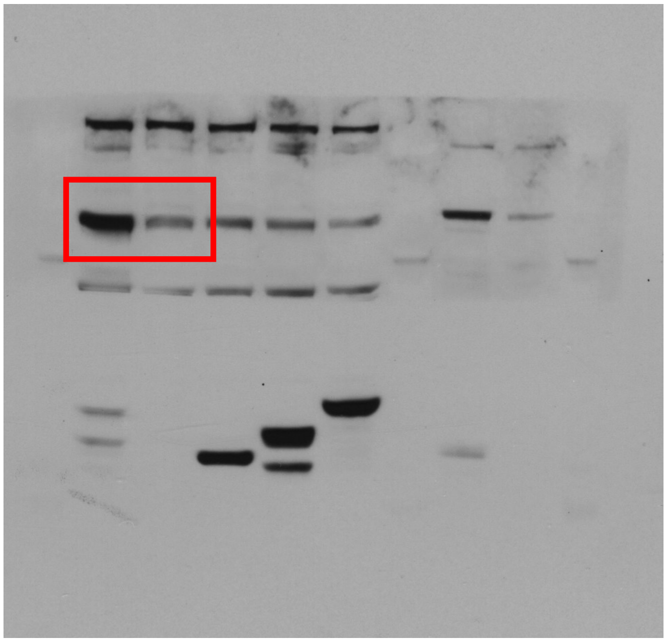

MondoA(MNT^{KO})

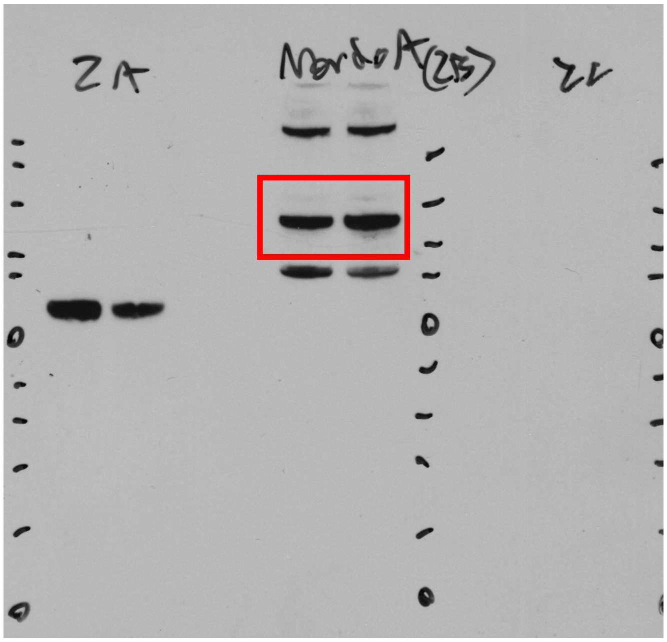

FIGURE 8E

MAX(MLX^{KO})

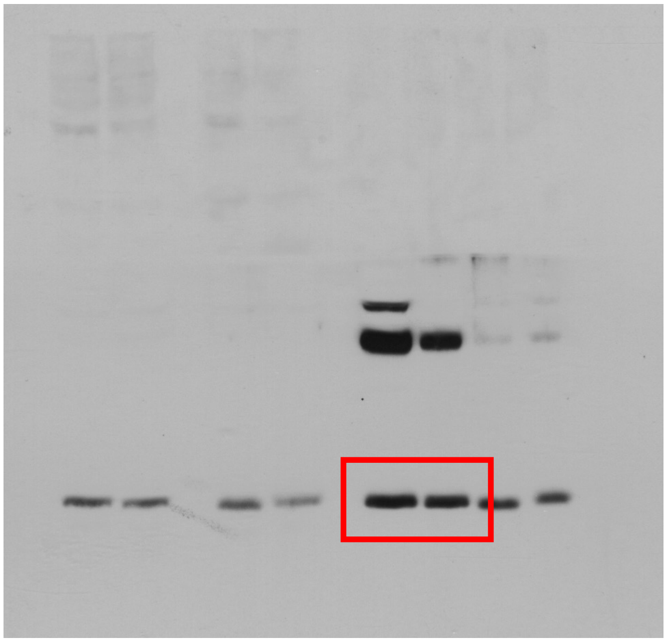

MAX(MNT^{KO})

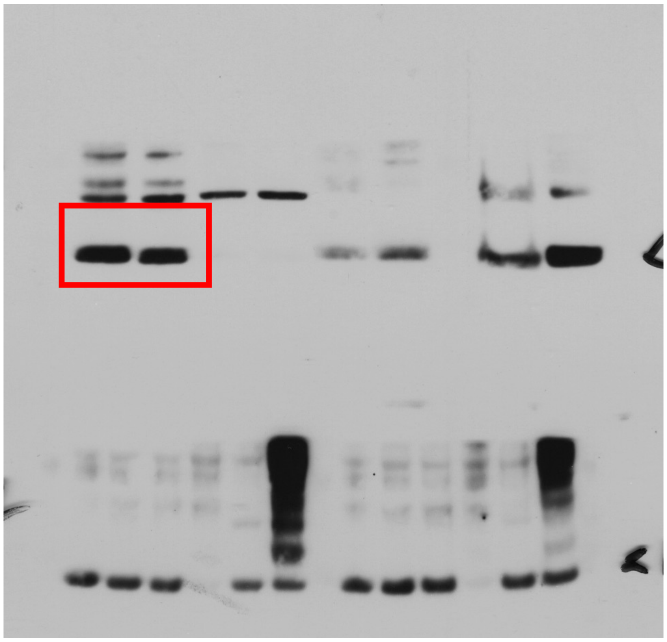

MNT(MLX^{KO})

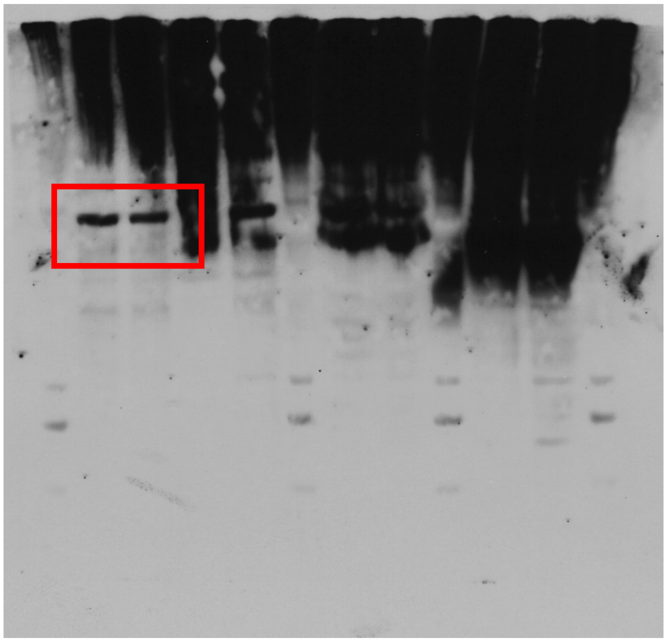

MNT(MNT^{KO})

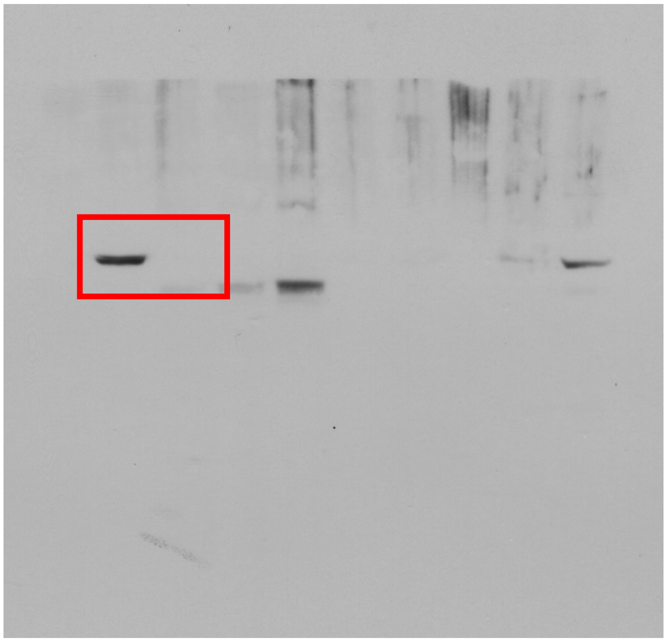

FIGURE 8E

TXNIP(MNT^{KO})
BIM(MNT^{KO})

TXNIP(MLX^{KO})
BIM(MLX^{KO})

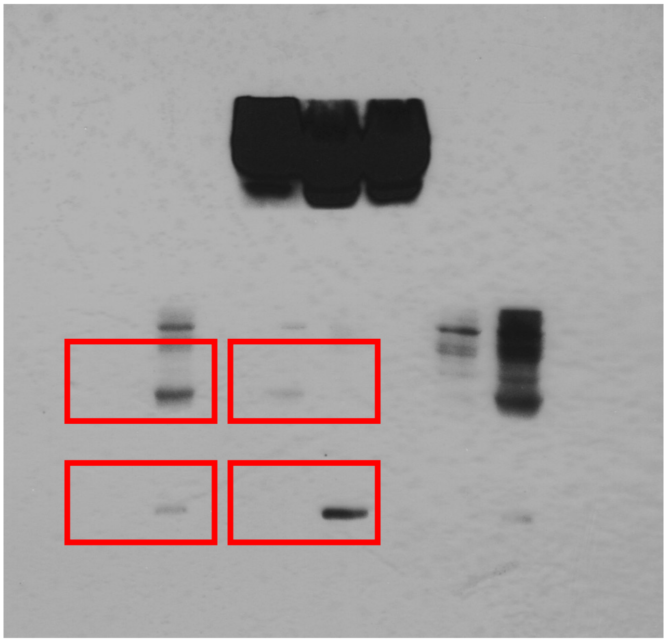

MYC

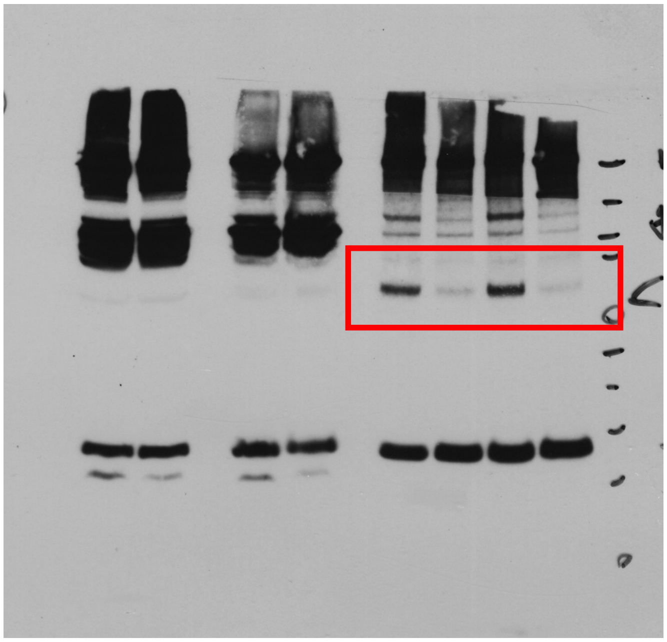

ATF4

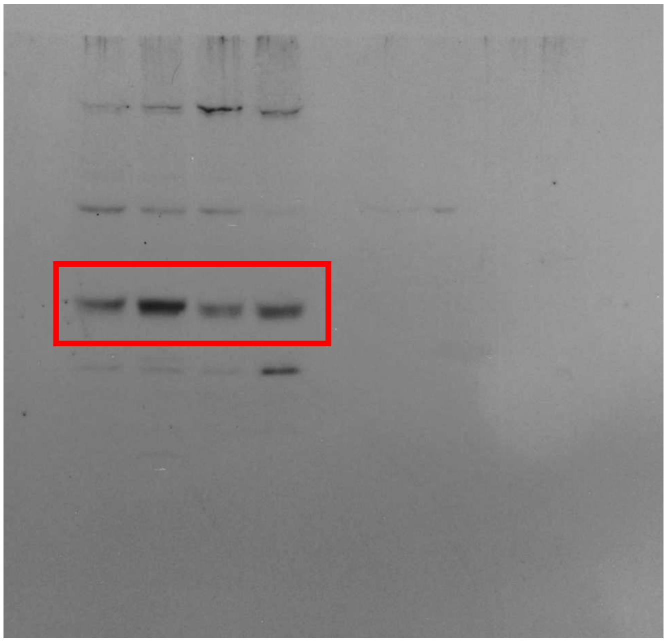

TUBULIN

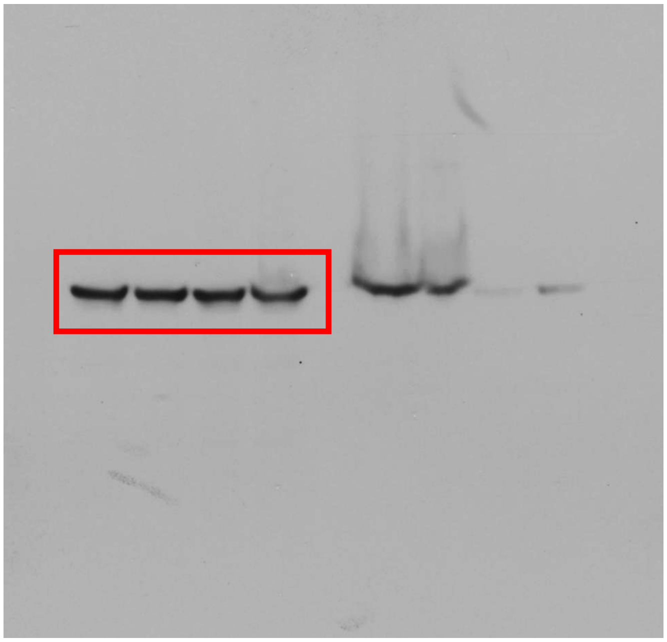

FIGURE 9C

MNT

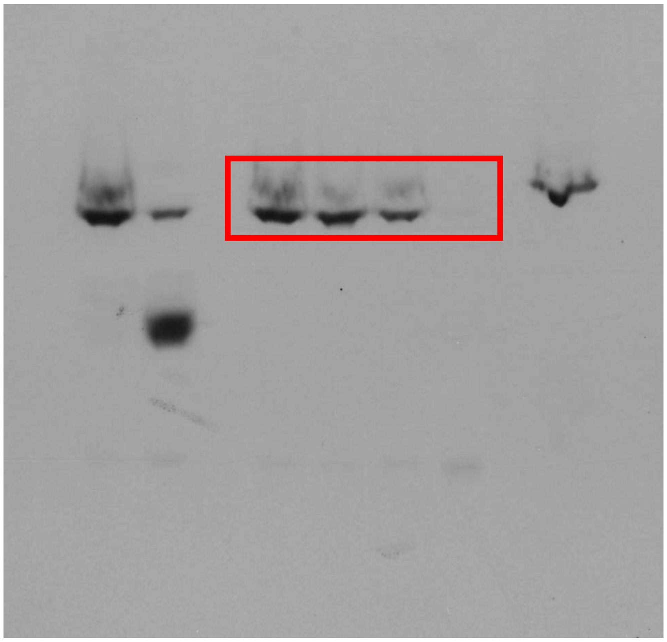

MondoA, TXNIP, MLX and BIM

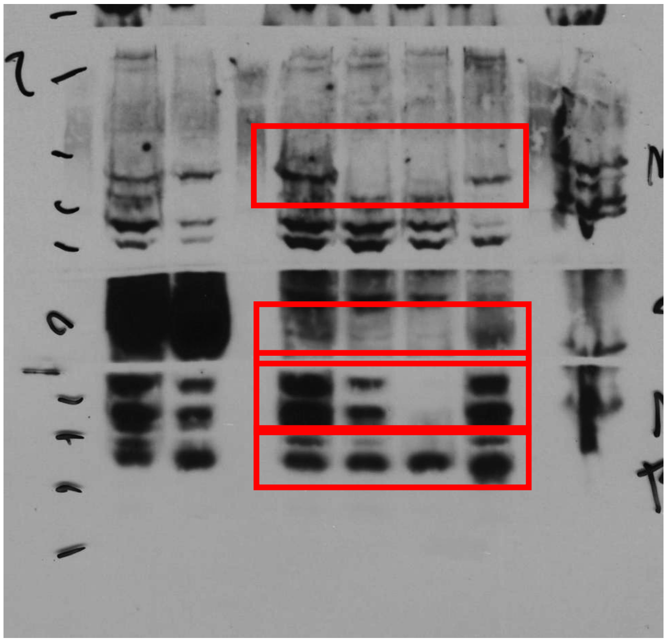

H2A

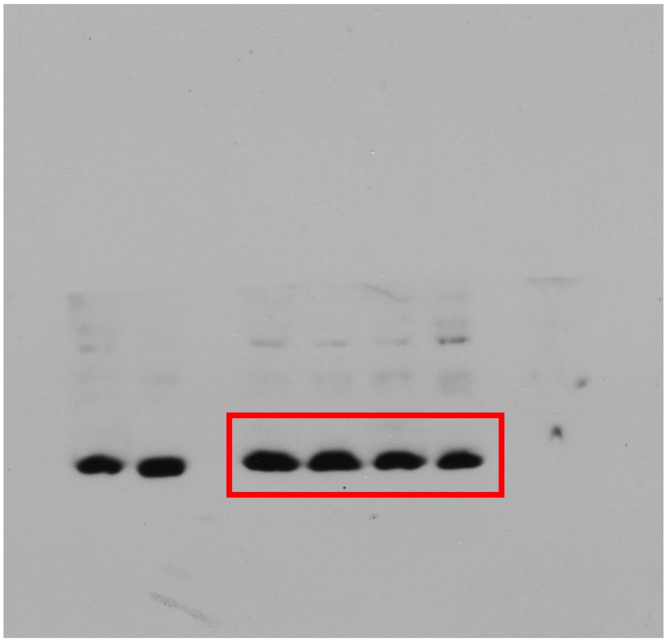

FIGURE 9E

PARP

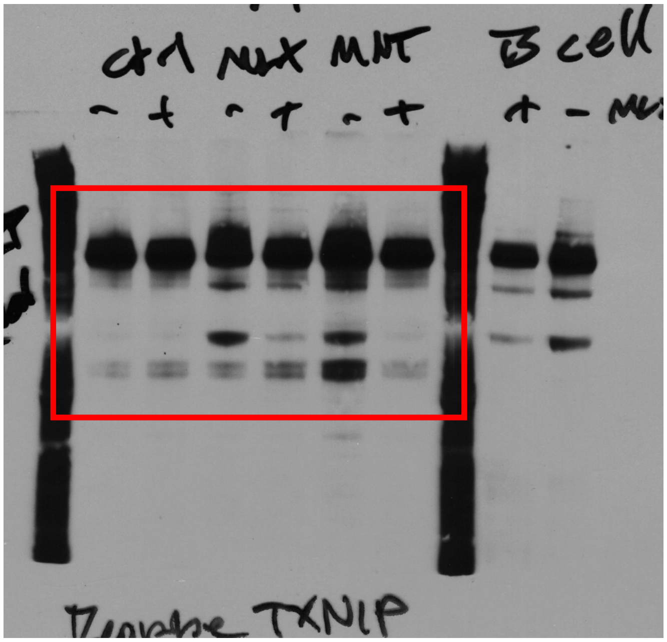

BIM

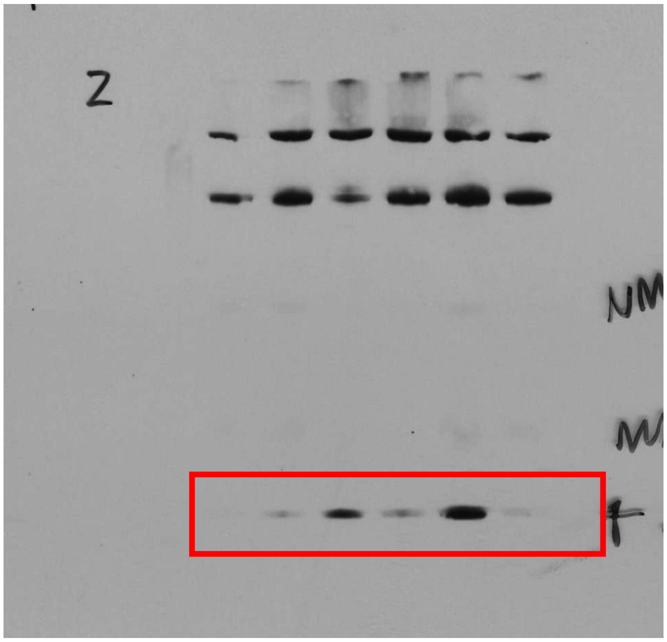

γH2AX

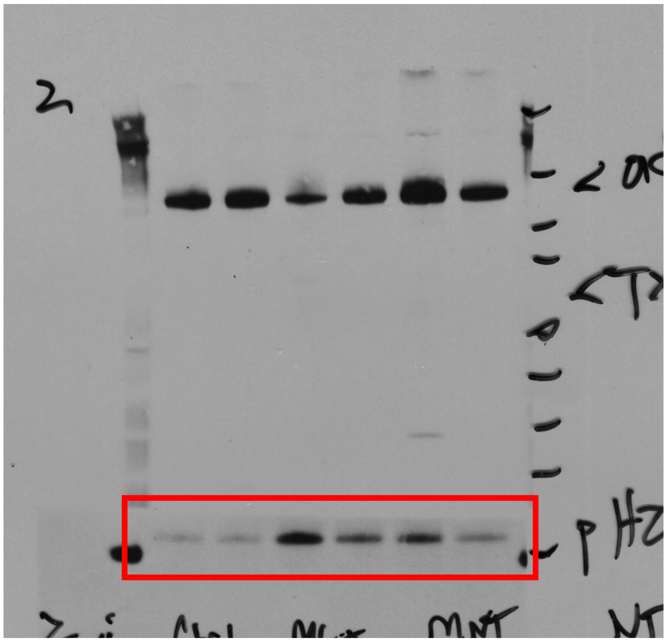

TUBULIN

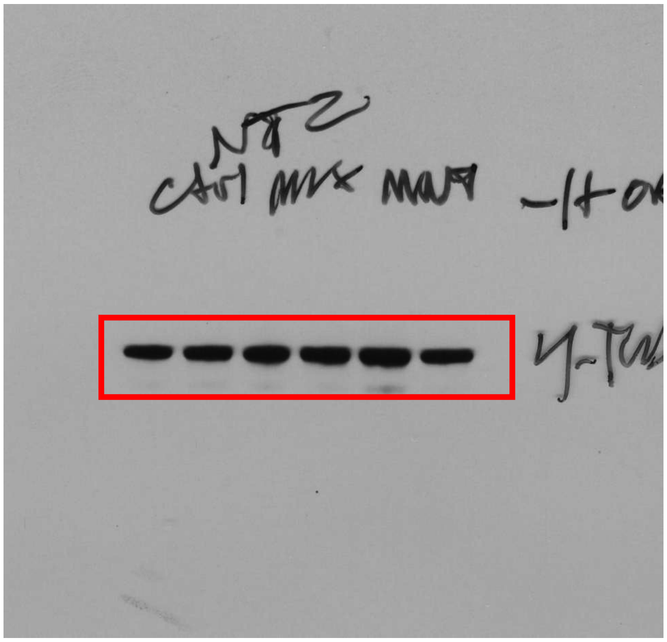

FIGURE 9F

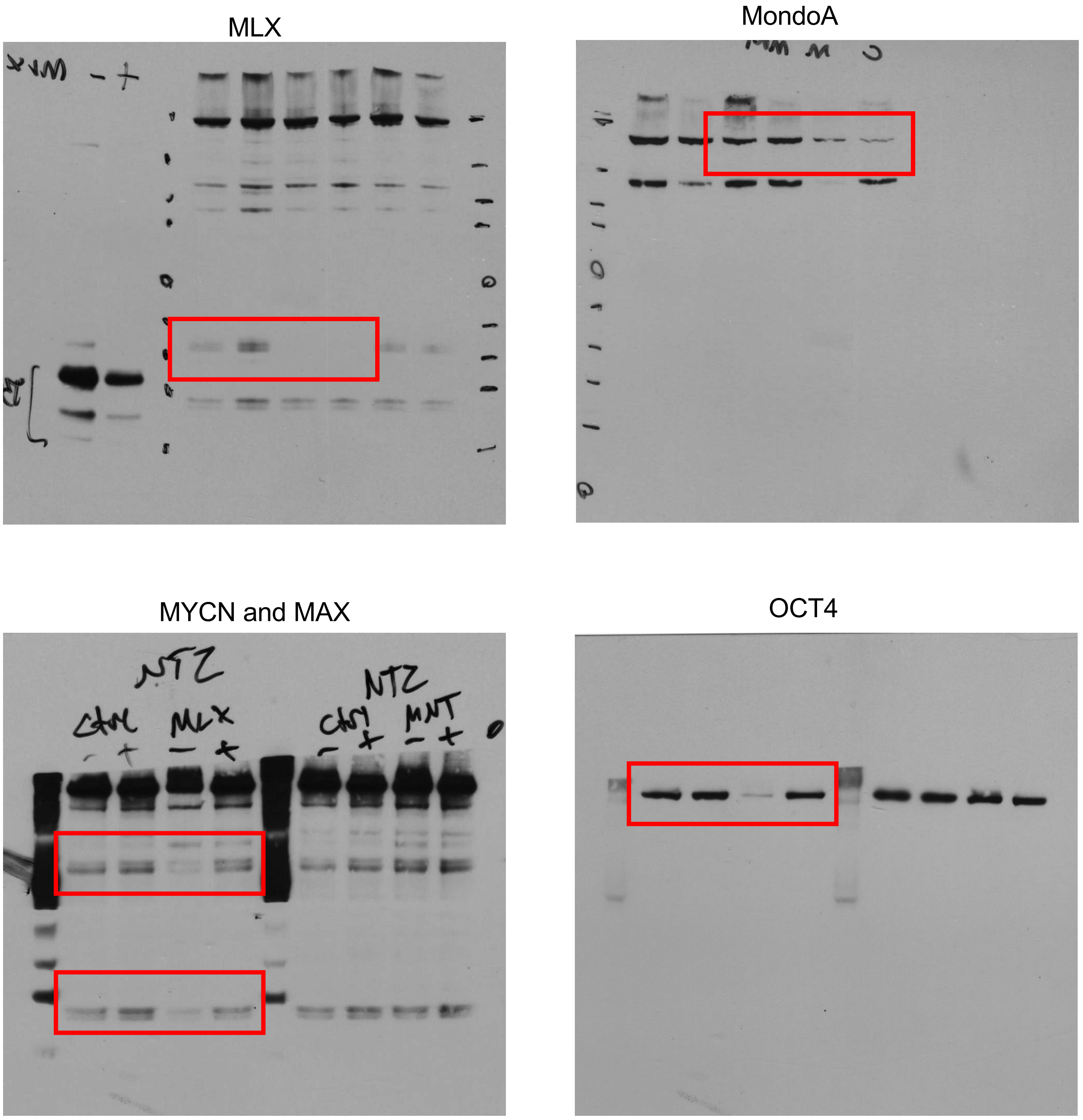

FIGURE 9F

FASN

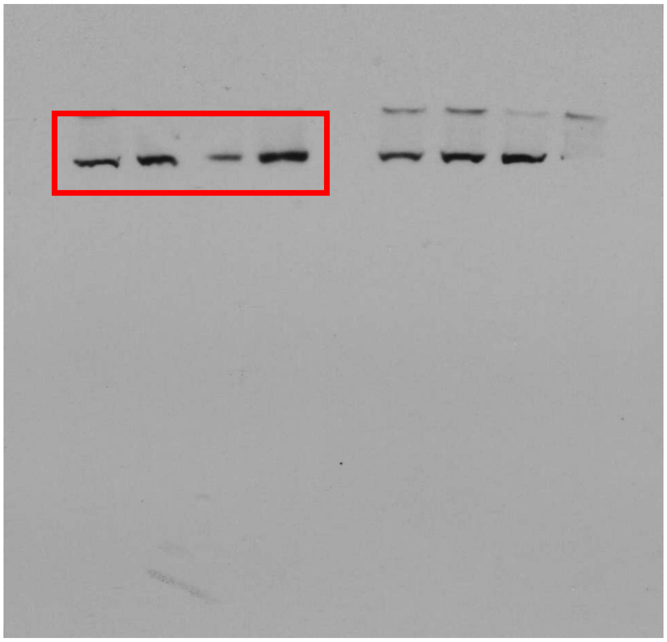

TUBULIN

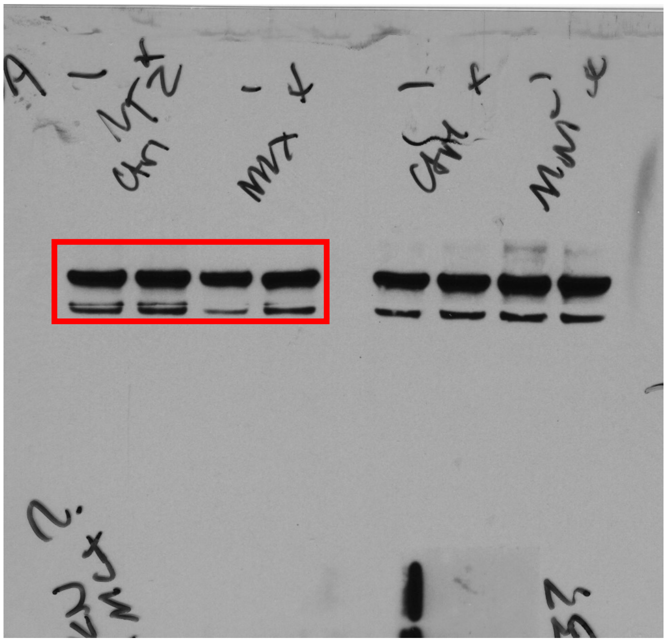

FIGURE 9G

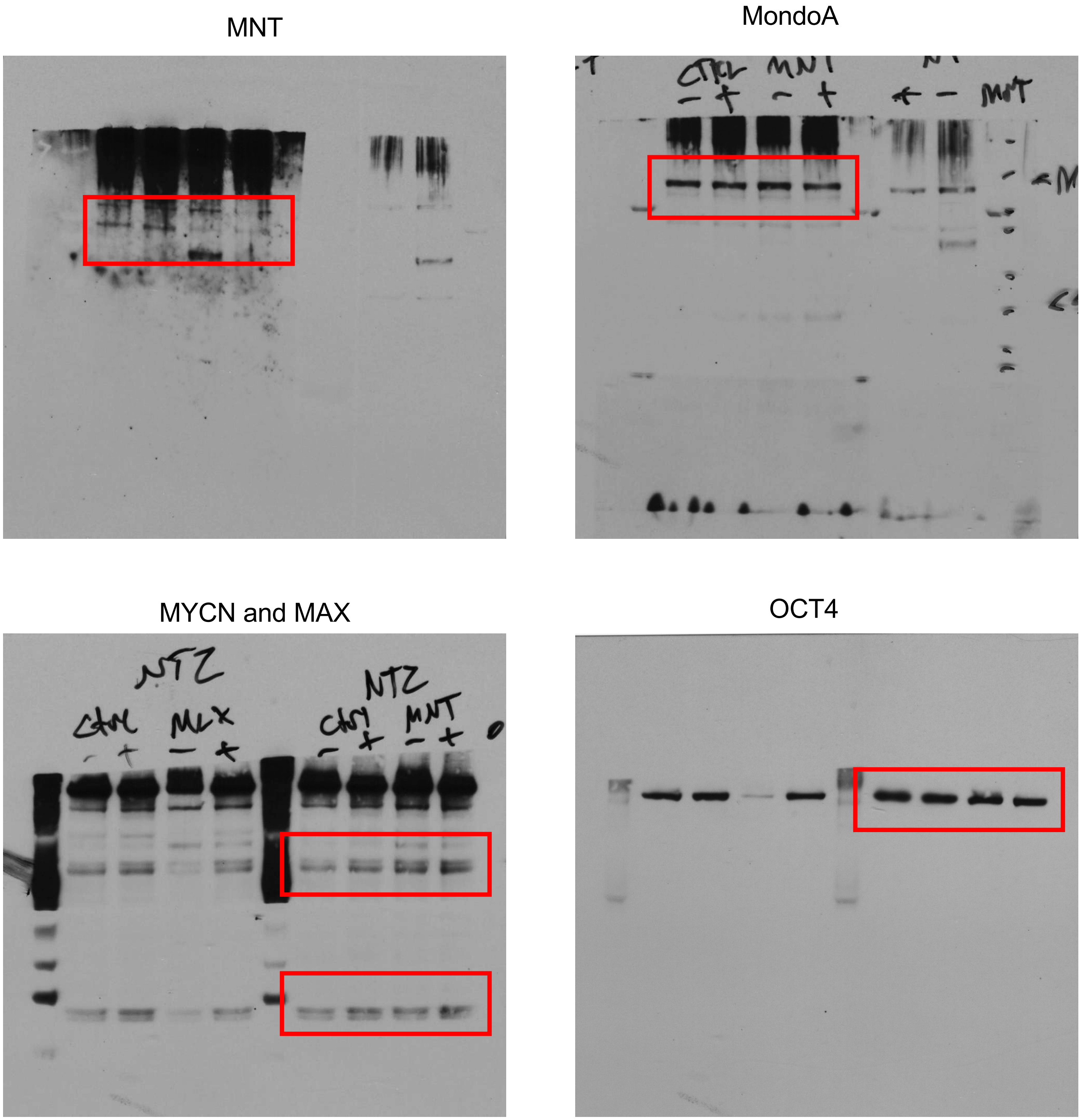

FIGURE 9G

FASN

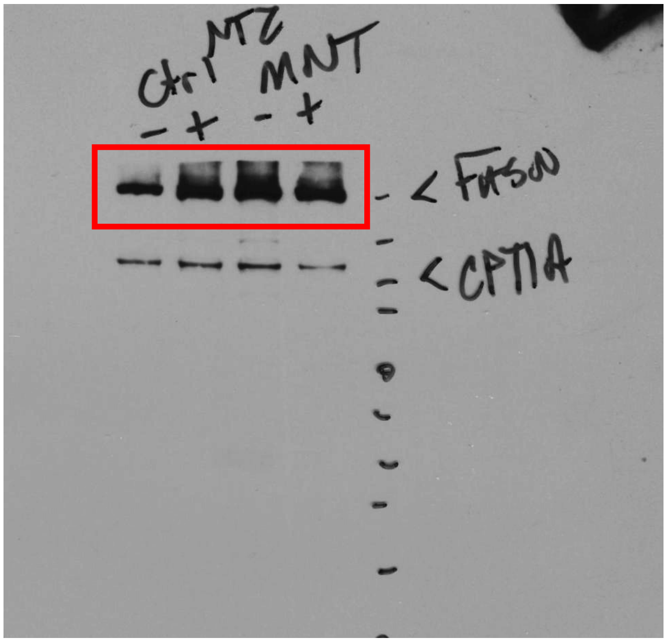

TUBULIN

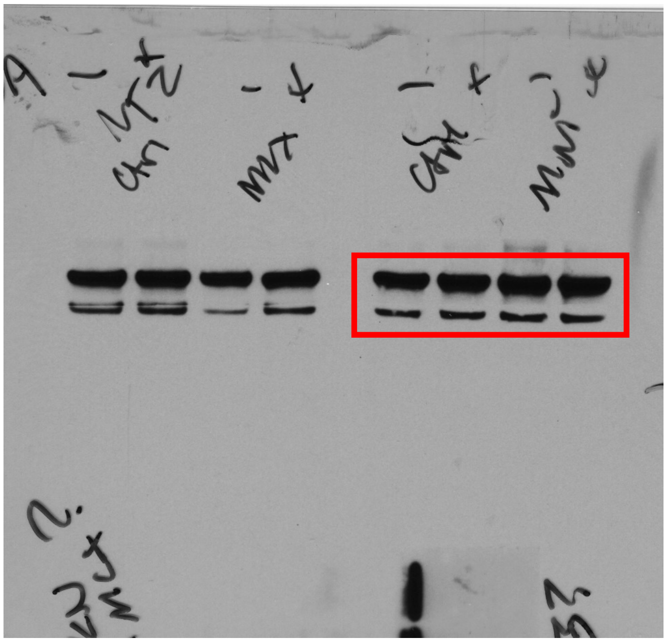

FIGURE S9B

MondoA, MLX and BIM

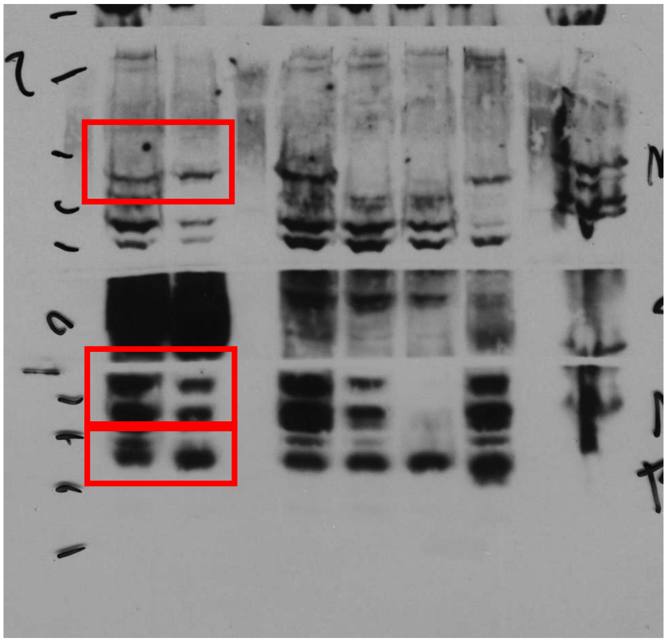

MNT

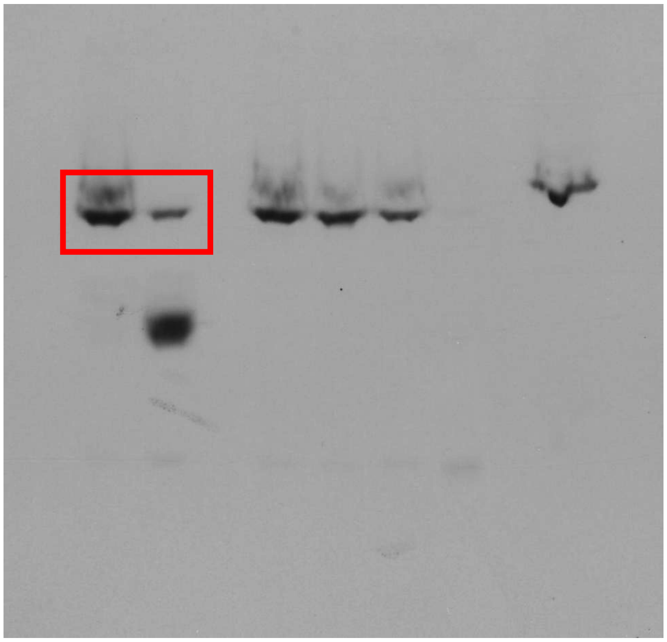

TXNIP

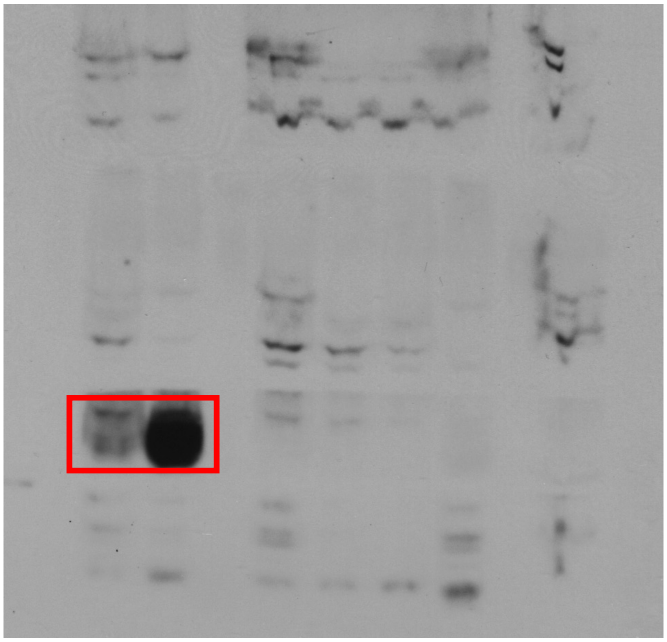

H2A

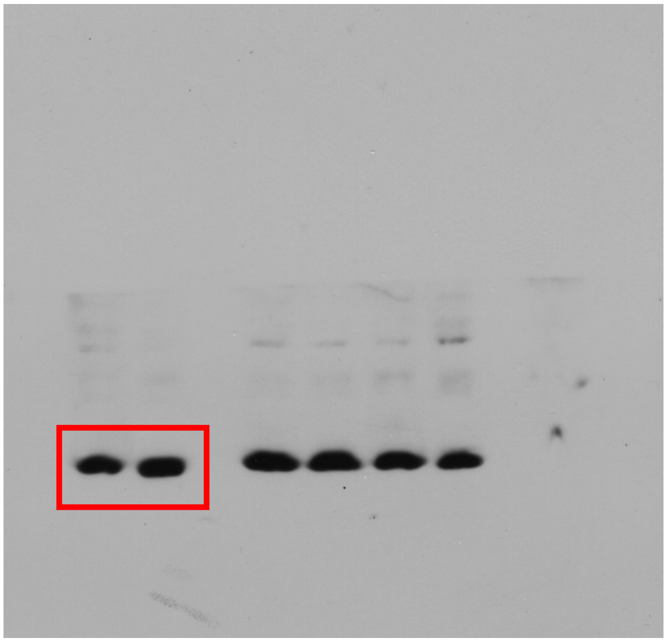

FIGURE S9C

MNT

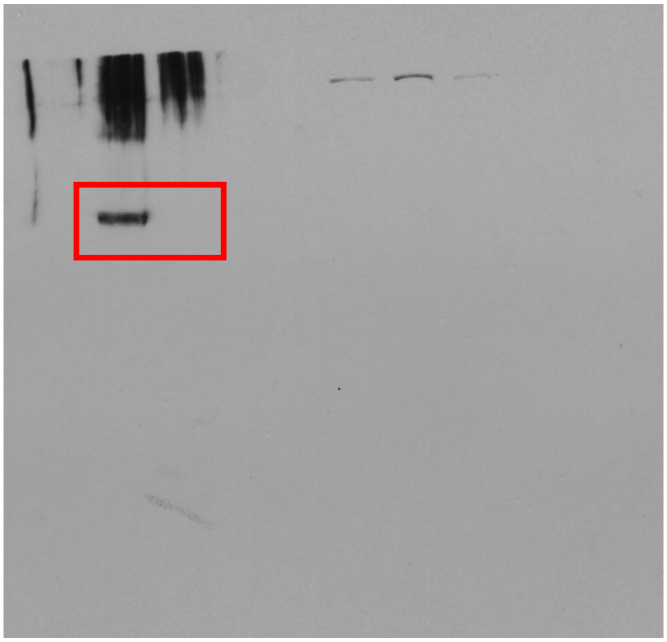

MLX

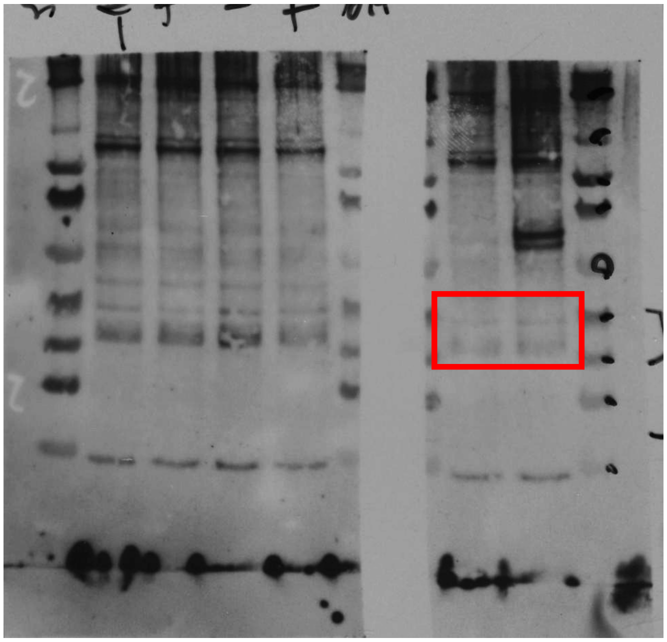

MondoA

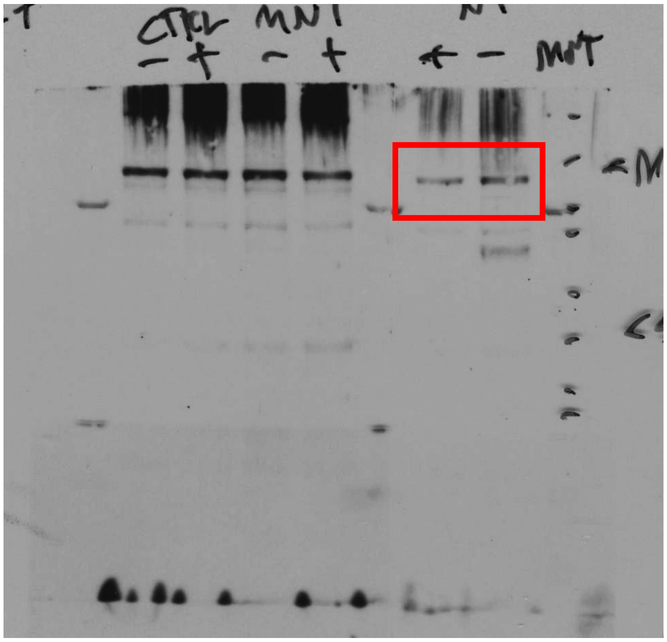

FASN

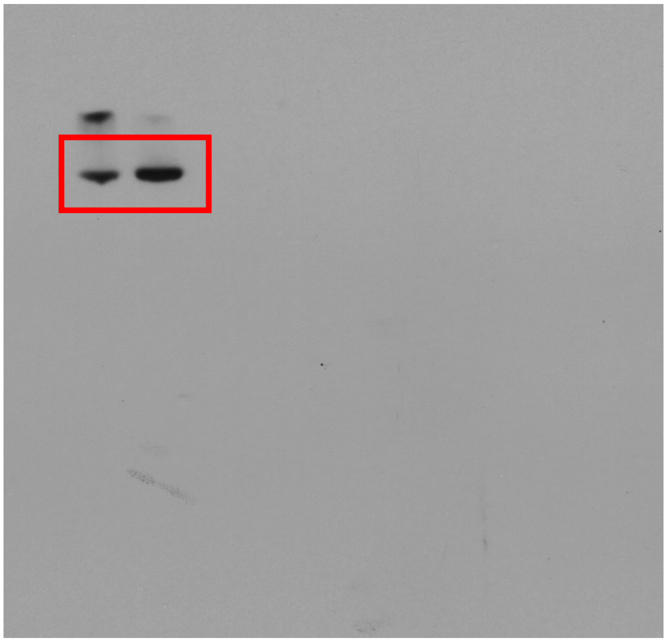

FIGURE S9C

SCD

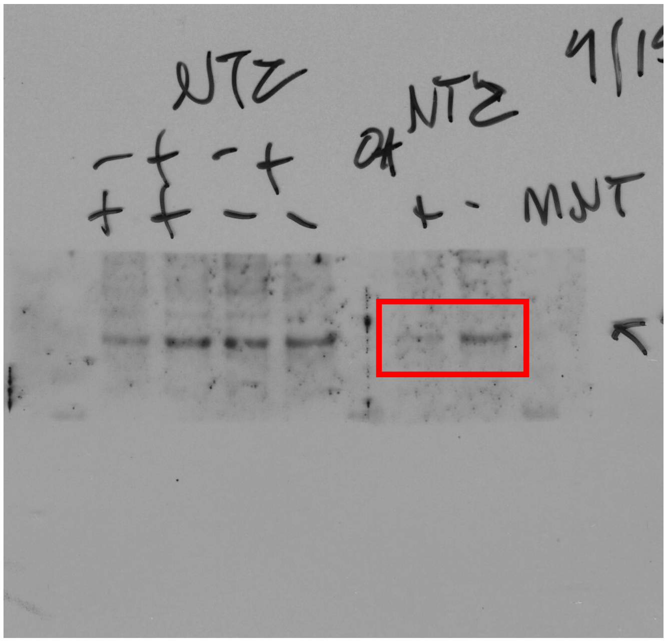

TUBULIN

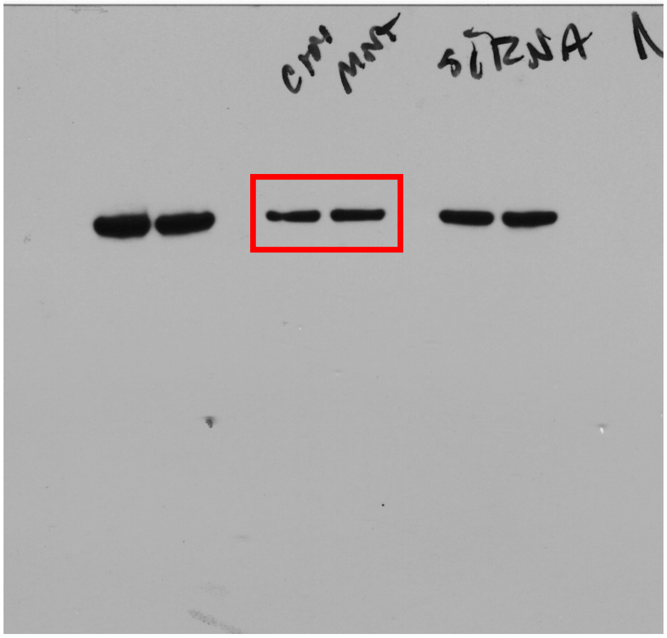

Supplement: S1 Raw Images — WB, western blot. (PDF) [file pbio.3001085.s012.pdf]
