# Supplementary material for: The glucose-sensing transcription factor MLX balances metabolism and stress to suppress apoptosis and maintain spermatogenesis
Source: PLoS Biol. 2021 Oct 20;19(10):e3001085. doi: 10.1371/journal.pbio.3001085 (PMC8528285; doi:10.1371/journal.pbio.3001085)
Supplement: Attachment — Submitted filename: Response to Reviewers_FINAL.docx [file pbio.3001085.s017.docx]

Response to Reviewers

We are grateful to the reviewers for their thorough reading and their thoughtful comments on our paper. In what follows we address the comments of the editor and each of the three reviewers. For ease in reading, we have indicated the reviewer comments in italics and numbered the comments where appropriate.

***Editor's Comment***

*You will see that the reviewers find your conclusions novel and interesting, but they also raise concerns with the degree of insight into the underlying mechanisms that mediate the observed defect in spermatogenesis. Specifically, we note the concern raised by Reviewer #2 regarding the specific cell types that mediate your observed phenotypes. This point has also been raised by the academic editor handling your submission, and we ask that you please include additional experimental data in your revised manuscript that addresses this (such as conditional knockout experiments).*

Our response to editor's comment:

As detailed below and in the revisions to the paper, we have directly addressed the issues concerning the cell types involved in our observed phenotypes. We demonstrate the widespread expression of MLX throughout the testes and provide further evidence that the stress response induced by loss of MLX is manifested at several stages of differentiation and in several cell types. This is consistent with our examination of a conditional knockout using Amh-Cre-targeted deletion of MLX in Sertoli cells, which we show contributes to the infertility phenotype. Moreover, in developing spermatozoa our data are consistent with a critical germ cell autonomous function for MLX in the proper completion and resolution of meiosis resulting in a failure to differentiate normally into mature spermatozoa. Our expression profiling and genome-wide binding analyses provide evidence that MLX normally functions in the maintenance of lipid and energy metabolism as well as in expression of genes specific to spermatogenesis which in turn is linked to attenuation of stress during spermatogenesis. These concepts are summarized in Revised Fig 10.

**Reviewer #1**

*Their in-depth functional studies revealed that germ cells in Mlx knockout mice are rather defective in the transition from round to elongated spermatids.
Furthermore, their comprehensive gene expression and ChIP-sequence analyses revealed that expression of important metabolic target and apoptosis-related genes are significantly down- and up-regulated in germ cells, respectively, mainly due to the loss of functions of MondoA-MLX and MNT-MLX complexes.

I consider that this is an excellent study that meets the high quality requirement for the journal. However, as described below, there are several portions that should be corrected in the paper.*

*The authors demonstrated in Figure 5A and Figure S6A, MAX binding sites are extensively altered due to the Mlx knockout in testis and 3T3 cells, respectively. I consider that the authors should conduct systematic bioinformatics analyses to characterize the Mlx-knockout dependent MAX binding sites. Are they enriched for stress and/or metabolism-related genes? Are they enriched for MNT-MLX binding sites? Is Atf4 gene one of such representative example? I assume this because Max signal shown in Figure S6B appears to be higher in MLX knockout than that of wild-type. I am especially interested in gene sets served as physiological binding sites for MLX (53 and 69 sites in testis and 3T*3 cells, respectively).

Our response to Reviewer #1 Point 1

The reviewer has brought up an interesting and relevant question that has prompted us to dig deeper into the nature of the shift in MAX binding sites. In the revised manuscript we have analyzed the MAX binding sites (referred to in the revised figures as MAX(MLX^KO^)-bound) that are observed upon *Mlx* deletion. Revised Fig. 4 (panels H and H') show volcano and violin plots relating to the expression changes in MAX(MLX^KO^)-bound loci indicating a trend towards downregulation. Focusing on the subset of MAX(MLX^KO^)-bound genes that are (i) de novo MAX binding sites (53 genes), (ii) differentially expressed in testis relative to WT, and (iii) relevant to testis biology, we identify the 9 genes listed in the blue shaded area in S4Table (and references therein). The genes in the list have been well established as being involved in apoptosis, stress response, lipid metabolism, MYC function, and Wnt signaling. Several of them have been previously shown to be required for male fertility. In addition, we carried out further enrichment analysis interrogating the CHEA transcription factor database, indicating that de novo MAX targets are also regulated by stress-related transcription factors (e.g., STAT3, P53, AR, WNT) (see revised S7 Fig) many of which were also identified in our GSEA analysis of differentially expressed genes upon MLX KO (Revised Fig 5 E,F,). We interpret these data to indicate that the cellular response to MLX loss involves a shift of the transcriptional regulatory machinery to genes that function in metabolism, stress response pathways and in apoptosis. We surmise that MAX binding to these genes contributes to the infertility phenotype we observe upon deletion of MLX. We have included this information in the revised text beginning at line 600 and in S4 Table. This table also lists genes bound by MLX and/or MAX or CREM and which are deregulated in MLX^KO^ testes, and also contribute to metabolism, stress and male fertility.

Regarding overlap with MNT: we find that more than half the genes in which MLX loss is balanced by a gain of MAX occupancy in 3T3 cells are also bound by MNT. Examining Enrichr analysis comparing MNT targets in wildtype vs. MLX deletion we find that MNT binding to ER stress related genes is decreased upon MLX loss, as shown in S8D-E, MNT only enriches for Unfolded Protein Response, amongst other stress pathways in MLX WT 3T3 (MNT(WT)-Bound ONLY). While it is unclear what precise role MNT is playing in terms of gene expression at these bound sites, several previous studies have shown MNT to suppress apoptosis in the context of dysregulated MYC and we anticipate that the altered binding of MNT would contribute to the decreased cell survival upon MLX loss.

In the case of Atf4, the reviewer is correct in noting that MAX occupancy appears to increase in the MLX^KO^. However, MNT occupancy does not significantly change at the Atf4 locus and we have also observed several MYC network and other factors associated with this gene without major alterations in ATF4 protein expression which is generally known to be largely controlled at the level of translation.

Minor comments:
1 (page 6 line 3 from the bottom) :
It is indeed possible to say that number of spermatozoa is decreased, but it is not possible to judge their mature levels only from the HE staining panels shown in Figure 1G.

Our response to reviewer #1 minor comment 1:

In the revised paper we have now included images of cauda spermatozoa (S1C Fig) as well as clarifying the text.

*2 (page 7 line 10 from the bottom) :
Ratios of round vs. elongated spermatids cannot be judged with Figure 1H.
If the authors judge the level of normality in morphology by the absence of DDX4 staining, I would recommend to cut and paste the sentences explaining about the meaning of DDX4 staining in epididymis described in page 9 line 11-14 in this portion.*

Our response to reviewer #1 minor comment 2:

We agree and have now included the information and reasoning for the DDX4 staining (Revised paper lines 226-228).

*3 (page 9 line 8 and other portions) :
Phosphor-gammaH2AX should be changed to gammaH2AX, since gammaH2AX means phosphorylated H2AX.*

Our response to reviewer #1 minor comment 3:

Thanks for this correction - we have made the appropriate changes throughout.

*Figure 3H, 3I and Figure S2C
Letters along with heat maps cannot read.*

Our response to reviewer #1 minor comment 4:

We have increased the resolution of all three heat maps: please see Fig 5 and S3C Fig.

*In Figure 3 legend:
Panels (A-K) in Figure 3 are appropriated cited in the text, but in Figure legend in which panels E, F and G are explained as a single panel (panel E).*

Our response to reviewer #1 minor comment 5:

We apologize for this oversight and have now revised with the correct figure legend. As requested by Reviewer #2 we have also reworked and clarified all the figure legends.

Reviewer #2

*1- The main finding of the manuscript (the role of Mlx/MondoA in male germ cell development) is novel and interesting, bringing a new dimension to the Mlx/MondoA literature. Moreover, the mouse model presented in this study will be of high importance to the field. The spermatogenesis phenotype is rather thoroughly characterized by histological stainings and specific markers, along with genomic analysis of targets of known regulators of male germ cell development. Moreover, the metabolic role and target genes of Mlx are characterized by state-of-the art metabolomics, genomics and further validated by Western blot analyses. However, despite the wealth of data, the main conclusions of the manuscript (as listed in the 'highlights') remain at a rather general level. The current version of the manuscript does not address sufficiently well, which target genes/cellular processes underlie the observed defects in spermatogenesis or which stage/cell type Mlx/Mondo is necessary for. In my
opinion, to be a strong candidate for a journal of broad readership, like Plos Biology, the study should yield more mechanistic insight.
For example:
-the rescue observed by oleic acid in teratoma cells serves as a promising starting point for deepening the analysis of the potential the role of Mlx/MondoA regulated lipogenesis in male germ cell development. Are their lipid-related phenotypes observed in the testis of Mlx KO mice or does interference of lipogenesis phenocopy loss of Mlx?
-immunostaining with Mlx-specific antibodies to analyze Mlx expression/localization in the testis would bring additional insight into the dynamics/cell type specificity of Mlx function.*

Our response to reviewer #2 point 1

In the revised paper we have stressed the fact that MLX is widely expressed in several cell types and at different stages of differentiation in the testis and that it is involved in the transcriptional regulation of a wide range of genes and pathways. This is consistent with the immunostaining data that we have now included in the revised paper at the request of the reviewer (see revised Fig 2A-B). We have also added new information by including recent data on our specific conditional MLX deletion in Sertoli cells by employing Amh-Cre (revised text beginning at line 241 and Fig 4 and S4). As described in the revised text MLX deletion in Sertoli cells results in infertility but only partially phenocopies our constitutive deletion of MLX. The partial effect of Sertoli cell Mlx deletion supports the notion that more than one cell type contributes to the infertility phenotype.

The reviewer points to our extensive metabolic analysis and our ability to rescue MLX depleted teratoma cells as indicative of the potential importance of lipid metabolism as a basis for the MLX related infertility. We completely agree, and in the revised paper have stressed this point (particularly our demonstration of a significant increase in acyl-carnitine species due to MLX deletion in the testis - see Fig 6E) and included citations for numerous previous studies that have linked lipid metabolism to normal spermatogenesis. Moreoever, genes that regulate lipid metabolism in testes are bound by MLX (S4 Table). Our data strongly point to increased stress and apoptosis as the major outcome due to MLX loss in the testis. Moreover, this is strongly supported by our gene expression and DNA occupancy studies which delineate a shift in transcription factor occupancy from lineage-specific genes to stress response and apoptotic genes upon MLX deletion. We feel strongly that, taken together, our studies lead to significant mechanistic insight into the role of the extended MYC network in regulating multiple processes during spermatogenesis.

*Reviewer #2 Point 2*

*The manuscript contains massive datasets obtained from various models/sources, some unrelated to testis (such as metabolomics of serum and genomic data from various unrelated cell types, e.g. primary B cells, 3T3 cells). This poses a challenge to coherent data presentation. It would also be helpful to have more explanatory figure legends to eliminate the need jump between results, figures and methods to understand the experimental setup and the main conclusions from the data. Therefore, I strongly encourage the authors to significantly edit the manuscript in a way that better helps the reader to follow the logic of the experimentation and think carefully, if all the data included into the current manuscript is relevant. The authors should make an effort to better walk the reader through the experiments and conclusions, keeping the focus on the main theme of the manuscript (Mlx in male germ cell development).*

Our response to reviewer #2 point 2

We take the reviewer's point here. The inclusion of other cell types was meant to illustrate the conserved and widespread functional roles of the MLX branch of the MYC network in terms of its role in stress response. We realize that inclusion of all the systems was indeed overwhelming. In the revised manuscript we have removed most of the B cell data (except for the sFASL killing assay as these are primary cells derived from the mouse), and all of the HepG2 cell data to simplify the content. We have worked to clarify the figure legends for all the included figures in addition to rearranging and rewriting several sections of the manuscript in both the Results and Discussion sections. We retained the 3T3 cell data as it directly relates to previous studies and, more importantly, is a system in which we could readily perturb and measure biological and genomic changes in vitro.

*Reviewer #2 point 3*

*Statistics:
The statistical data, indicated by stars, should be displayed in a way that it is clear, which samples are being compared. The statistical test should be mentioned in the Figure legend.

Minor:
Supplemental Figure 7D contains eight 'OE -/+' but only six bars.*

Our response to reviewer #2 point 3

These changes have been made. In the revised manuscript figure legends we have added information on the statistical tests employed in every experiment shown. The initial data used to generate the plots is provided in the Supplementary data material. Note this in the revised manuscript this information is now in S9D Fig.

*Comments of Reviewer #3*

*Reviewer #3 (Ricardo Daniel Moreno Mauro): The manuscript entitled "MLX balances metabolism and stress to suppress apoptosis and mantain spermatogenesis" by Carroll et al.,  gives a nice pack of information about the role of  this transcription facor using up-to-date-technology. However, according to the criteria of this reviewer there are some issues that need to be addressed*

*Reviewer #3 point 1
Results
1.- I think the best controls are heterozygous and not wild type mouse, as the authors use in the manuscript.*

Our response to reviewer #3, Point 1

There are certainly some contexts in which comparison with homozygous deletion to heterozygous deletion is warranted, however we do not think that this is the case for the MLX KO mice. While we show in Figure 1 (Mating tests) and Supplemental Figure 1 (histology) that heterozygous animals appear to be equivalent to wildtype we cannot, at this stage, rule out haploinsufficiency at some level that could potentially affect multiple aspects of the analysis. and Given our previous extensive experience with developmental analysis of the MYC network in mice and because this is the first genetic analysis of MLX function, we feel confident that comparison with WT is the best approach. Future studies will likely analyze the hets in more detail.

*Comments of reviewer #3, point 2*

*2.- Througthout all the manuscript authors use a wide range of different words that induce to confusion. Germ cells is a term generally use to refer spermatogonia, spermatocyte and spermatids. After they leave the testis is better to name them spermatozoa.*

Our response to reviewer #3, Point 2

We appreciate the reviewer pointing this out and have now gone through the paper and made the appropriate nomenclature changes.

*Comments of reviewer #3, point 3*

*3.- Figure 1C-F: Are the same mice in all these figures? Why in some graphs authors show 5 spots (E) while in other 7 (F)?*

Our response to reviewer #3, Point 3
Seven mice were used for the body and testis weights, and 5 mice were used for the DSP, epididymal and seminal vesicle weights. The figures show the complete set of data from these groups, and no data points were omitted from the figures shown.

*Comments of reviewer #3, point 4*

*4.- Figure 1G. Authors shows abcence of spermatozoa in t MLK-KO mice epididymis. Could this be due to a slowdown in epididmis transit from head to cauda? The authors need to examine all the three major segments of the epididmys to rule out this option.*

Our response to reviewer #3, Point 4

**The reviewer raises an interesting point. We have supplied multiple images of different epididymal segments. We observe the sloughed cells throughout and never observed a buildup in any segment, consistent with transit from the caput to cauda. Interestingly, our IF shows MLX present in the epididymal epithelium, so it could play a role, but that would not explain decreased Daily Sperm Production rate in the testes.**

*Comments of reviewer #3, point 5*

*5.- What the authors mean by "testis and epididymis tissue were disrupted"? Abnormal tissue histology?*

Our response to reviewer #3, Point 5

Yes, exactly. The text has been updated to reflect this correction.

*Comments of reviewer #3, point 6*
*6.- Could the authors define what they mean by abnormal  and acellular seminiferous tubules?*

Our response to reviewer #3, Point **6**

We are referring to the tubules largely devoid of germ cells that we observed and quantified in testes of MLX KO mice. We now add data showing that these tubules lack germ cells but maintain expression of SOX9, a Sertoli cell marker (Fig 4A). We have added more images and quantification in S4A-C Fig.

*Comment of reviewer #3, Point 7*

*7.- I suggest a depper morphometric study in these samples (e.g area and diamter of seminiferous tubules)*

Our response to reviewer #3, Point 7

We have not carried out a morphometric analysis but have now further characterized and quantitated acellular tubules in WT and MLX^KO^ mice and compared them to Sertoli-specific Amh-Cre+ and floxed mice (see Fig S4C). We show that the abnormal tubules are not solely a result of MLX loss in Sertoli cells. Suggesting germ cell apoptosis is responsible for the loss of cells.

*Reviewer #3 point 8
8.- Authors mention that MLK-KO mice present oligoastheteratozoospermia (OAT). However they do not show that mature cauda epididymal spermatoza have morphological abnormaligies.*

*Their data by flow cytometry only suggest that some round spermatids reach the epididymis, but this is not a proof of tetratozoopermia. Please see these examples ( Mol Hum Reprod . 2021 Jan 22;27(1):gaaa083. doi: 10.1093/molehr/gaaa083; PLoS One. 2013 Nov 28;8(11):e80607. doi: 0.1371/journal.pone.0080607. eCollection 201). On the same token, Figs 2C, D, H show a reduction in %normal motility in MLKKO males. What is normal motility? Asthezoospermia means a reduction in progresive motility.*

Our response to Reviewer #3 point 8

We thank the reviewer for pointing this out. In the revised paper we show examples of the morphological abnormalities seen in cauda epididymal spermatozoa (S1C Fig). We interpret our observations to indicate that MLX loss shares certain features with OAT and have modified the text to indicate this (see Discussion). The 2021 Mol Hum Reprod paper cited by the reviewer defines OAT in terms of abnormal sperm morphology, low sperm count and poor sperm motility. We assessed the head shape, acrosome, midpiece and tail (shown in Fig 2D) of the (cauda epididymal) sperm. We did not tabulate non-sperm cells in the epididymis. For the motility measurement we measured progressive motility, scored for spermatozoa from the caudal epididymis.

*Reviewer #3 point 9*

9.- Figure 3K needs propers antibody controls since the label looks quite non-specific.

Our response to Reviewer #3 point 9

In the revised paper we have included IHC as well as IF controls where appropriate. For MLX, we have validated the signal by using MLX^KO^ tissue. See Fig 2A, Fig 5J,K, S4D Fig.

*Reviewer #3 point 10*

*10.- Why authors compare their date in mice with samples from teratozoospermic patients, since they suggest that MLK_KO mice are oligoasthenoteratozoospermic?*

Our response to reviewer #3 point 10

Our point here was to show that genes bound and regulated (directly or indirectly) by MLX are affected in a human spermatogenesis disorder. We were not able to find an equivalent data set for OAT available in the GEO.
