# Supplementary material for: The glucose-sensing transcription factor MLX balances metabolism and stress to suppress apoptosis and maintain spermatogenesis
Source: PLoS Biol. 2021 Oct 20;19(10):e3001085. doi: 10.1371/journal.pbio.3001085 (PMC8528285; doi:10.1371/journal.pbio.3001085)
Supplement: Attachment — Submitted filename: Response to Reviewers.docx [file pbio.3001085.s018.docx]

Response to Reviewers

We thank the three reviewers for their highly positive remarks on our revised manuscript.

In response to the editor's suggestion, we have modified the title of the paper: *The glucose-sensing transcription factor MLX balances metabolism and stress to suppress apoptosis and maintain spermatogenesis*

Reviewer remarks:

Reviewer #1:
The authors addressed all my concerns appropriately and I have not any further comments other than recommending the publication of this manuscript in PLoS Biology.

*Our response: None required*


Reviewer #2:
The authors have addressed my original concerns very thoroughly and the manuscript is significantly improved. I am happy to recommend it to be accepted to Plos Biology. Before publication, please check the subheading "MNT and MAX transcription factors bind metabolic and stress targets shared with MAX" (lines 641 and 642).

I wish to congratulate the authors for this groundbreaking study.

*Our response: We have corrected the typo on lines 641-2 as pointed out by the reviewer.*


Reviewer #3 (Ricardo D. Moreno, signs his review):
I think the authors have made a great work. I have no further questions.

*Our response: None required*
